# Studying gastrulation by invagination: The bending of a cell sheet by mechanical cell properties using 3D deformable cell based simulations

**DOI:** 10.1371/journal.pcbi.1013151

**Published:** 2025-06-25

**Authors:** Roland M. Dries, Kim Y. Renders, Jaap A. Kaandorp

**Affiliations:** 1 Department of Bionanoscience, Kavli Institute of Nanoscience, Delft University of Technology, Delft, The Netherlands; 2 Computational Science Lab, University van Amsterdam, Amsterdam, The Netherlands; Utrecht University, NETHERLANDS, KINGDOM OF THE

## Abstract

Studying the bending of a cell sheet in vivo, like invagination in embryos, can be complex due to a multitude of cellular processes and properties that interact with each other. Computer simulations can help to unravel this process. 2D computer simulations, however, lack the ability to take into account the effect three-dimensional properties, like endodermal plate shape and cell number, have on the shape of an embryo. Therefore, we developed a 3D cell-based model, that is able to simulate cells as separate deformable entities with a conserved cell volume. A blastula is formed by adhering the cells together as a sphere. The simulation results showed that changing individual mechanical properties, like cell stiffness, cell-cell adhesion, and the apical constriction factor, had a direct effect on the cell’s behavior and future shape. These properties influenced the ability of a cell sheet to bend and eventually change the global shape of the embryo. The observed shape transitions the endodermal region goes through during the inward bending of the cell sheet in the simulation, can give an insight into the mechanisms involved, and timing of events in biological model organisms. Changing geometrical properties (endodermal plate shape, endodermal cell number and the start position of constriction), which is not possible in 2D models, showed that the inwards bending is more dependent on the number of cells involved than on the shape of the endodermal region, and thus that the invagination process is very robust to irregularities. When qualitatively comparing our simulation results to biological data from literature, we saw that our simulations did not exactly reproduce the shapes observed in nature. This might indicate that additional mechanisms are playing a role during invagination.

## Author summary

Organisms that develop from a fertilized egg into a complex multicellular organism, go through multiple shape changes. One of the first changes that occurs, takes place during gastrulation, where a part of the embryo bends inward to form the germ layers, creating a multilayered embryo. Examining how this process takes place and which mechanical properties are necessary to change the local cell shape tells us something about the development of an organism and how robust the process is. We used a computer model to simulate a 3D embryo with cells that can deform and adhere together to investigate the bending of a cell sheet. Locally deforming a cell that is connected to other cells transfers this deformation to the surrounding cells. Thereby transforming a part of the embryo. The number of cells and the pattern formed by the interconnected cells determine the embryo’s shape. 3D simulation outcomes differed from real embryos, suggesting that there are more mechanisms necessary for full gastrulation, that are not captured by 2D simulations.

## Introduction

**Fig 1:**
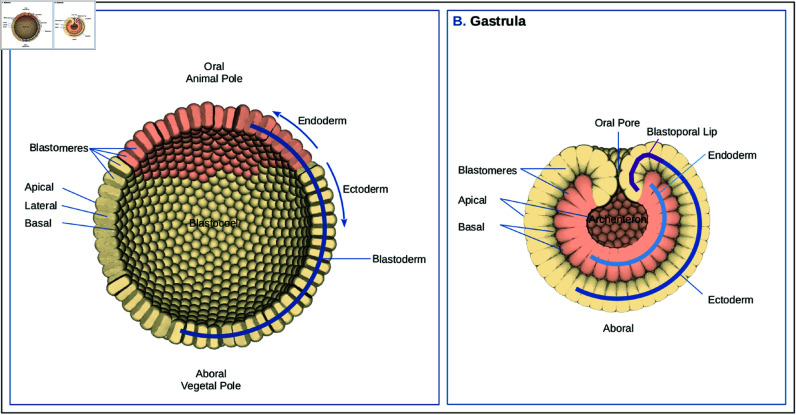
Blastula and gastrula illustration. **(A) Blastula**. Illustration of a blastula of a diploblastic organism. The endodermal plate (approximately one fourth of the total number of cells) is located on the future oral side (animal pole) indicated with salmon. Opposite, is the aboral (vegetal) pole. The axis between the poles forms the primary oral-aboral axis. The rest of the blastoderm is formed by the ectodermal cells (colored beige), that together with the endoderm encloses the blastocoel (internal space). The epithelialized cells (blastomeres) are laterally adhered together, forming the spherical hollow blastula. The apical area of the cells is directed outward, while the basal area is directed toward the blastocoel. **(B) Gastrula**. Illustration of an invaginated gastrula. After invagination the endodermal plate has moved into the blastocoel and aligned with the ectoderm, forming two germ layers. The salmon colored cells form the endodermal layer that enclose the archenteron (primitive gut). The ectodermal layer forms the outside of the embryo. Where the ectodermal layer has curled into the opening, it forms the blastoporal lips (future pharynx), that enclose the blastoporal (oral) opening. The blastocoel has completely disappeared due to the basal alignment of the two cell layers.

During embryogenesis, it is the interplay of cellular activities that is responsible for the emergence of embryonic tissue shapes. The cell as an entity, has a central role in the development of morphological changes by changing its mechanical properties over time [1–4]. Which properties change and under what conditions, is under continuous investigation by looking at different model organisms (in vivo) [4–11] and computer simulations (in silico) [12–21]. An example of a morphological event that has been extensively studied, is the transition from blastula to gastrula, called gastrulation. Gastrulation is one of the earliest shape-changing processes during an animal’s morphogenetic development [1,11]. The blastula, a simple multi-cellular structure, that consists of an epithelialized cell layer, is actively transformed into a gastrula with different germ layers (mesoderm, endoderm and ectoderm). Eventually this embryo grows into a complex and organized multilayered organism with distinct tissue types [1,2,22,23]. There is not a single way for organisms to gastrulate. Different species have found different mechanisms, of which invagination is one [2]. During invagination, a group of cells, or several groups of connected blastula cells (blastomeres) that form an epithelial sheet, bend inwards. When the infolding layer aligns and connects with the remaining part of the blastula, it forms, in the case of diploblastic animals (2 germ layers), a double-layered embryo [2] (Fig 1).

**Fig 2:**
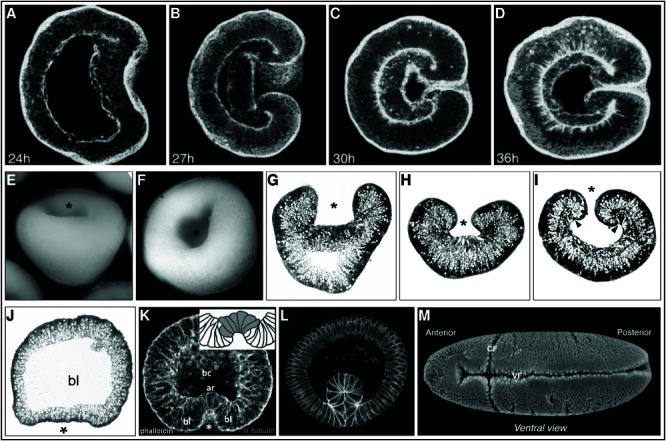
Biological images of invagination. (**A–D**) Confocal z-stacks of *Nematostella vectensis* invagination. The images show cross sections of phalloidin stained embryos of *Nematostella vectensis* at different developmental stages during gastrulation (time of development in hours in left bottom corner). The endodermal plate is directed to the right. (A) The endodermal plate starts to constrict. (B) Invagination continues, the endoderm first aligns laterally and moves aborally. (C) The endodermal and ectodermal layers are fully attached. The embryo has become spherical with a closed off blastoporal opening. (D) After alignment, the endoderm has spread out and reduced its height. Images modified from [31]. (**E–I**) *Favites abdita* (Stony coral) invagination. The images show invaginating *Favites abdita* embryos. (E), (F). Embryos, with oral view of the roundish blastoporal opening, indicated with an asterisk (*). (G). Cross section showing mid-gastrula stage. (H). Cross section showing a fully invaginated embryo with a bowl-like shape and reduced blastoporal opening. (I). The embryo has become more spherical again, and the endodermal layer has reduced its height. Images modified from [9]. (**J** and **K**) Initial invaginating plate shapes. Cross sections through invaginating embryos. (J). Flat invaginating endodermal plate shape seen in stony coral (*Dipsastraea (Favia) speciosa*). The asterisk (*) indicates endodermal plate. (K). Concave invaginating plate shape seen in *Aurelia aurita* embryo. bl=blastoporal lip. The asterisk (*) indicates the invaginated endodermal plate. Image (J) modified from [9], Image (K) modified from [32]. (**L** and **M**). *Drosophila* ventral furrow invagination. (L). Cross section through a *Drosophila* embryo and (M). Ventral view of *Drosophila* embryo. Images (L) modified from [33] Images (M) modified from[34].

The initial invagination area (plate) in the blastula can become flat or concave (Fig 2J and 2K) during the invagination process. This bending of an epithelial sheet, as seen during invagination (e.g., in fruit fly mesoderm cells invaginate, Fig 2L and 2M [6], and in Cnidaria (Starlet sea anemone, Fig 2A–2D, and Stony corals, Fig 2E–2I), endodermal cells invaginate [9–11]), can be accomplished when enough single cells change their shape from columnar to wedge shaped [24]. The cell deformations are caused by changes in the cell cortex, consisting of the actomyosin and microtubule cytoskeleton [23,25]. When cells actively constrict their apices and are confined by neighboring cells, the rest of the cell will passively change its shape. Depending on the circumstances, the cytosol and nucleus are displaced, resulting in local cell shape changes [26] like elongation [23,25] or expansion of the basal area, changing the shape to wedge, squad or bottle shaped [12]. Apical constriction has also been proven to be a strong driver of morphological changes in other organisms: blastopore lip in *Xenopus leavis* (Clawed frog), primitive streak in chick, and neural tube in mouse [27]. Constriction can be in a purse-string like fashion, where the apical surface becomes smaller but remains convex, as seen in *Nematostella vectensis* [10–12], or by apical flattening, as seen in *Drosophila melanogaster*, *C. elegans* [11], and *Clytia hemisphaerica* [28]. Through adhesion molecules (adherens junctions), which are located apical-lateral [23], the cortical tension (the force generated by apical constriction) [29] is passed on to neighboring cells [23]. This eventually causes the entire cell sheet to bend [24], changing the global shape of an organism. In turn, the mechanical and geometrical properties of the cell’s surroundings influence the internal structure of the cell, its shape, and its movement [30]. Embryo morphogenesis eventually comes down to local cell shape changes, which all depend on the mechanics of the individual cell [26].

Therefore, to understand the morphogenetic process of an organism, the first step is to understand the underlying mechanics of how a cell changes its shape [26]. In vivo research uses microscopy to look at apical constriction. But analyzing how local cell shape changes and forces created by the actin cytoskeleton affect the organism’s global shape is still difficult in vivo, since the cytoskeleton is involved in a lot of morphogenetic processes in the cell [1]. Another way to gain insight into complex mechanical processes is through computational simulations. Since we want to simulate invagination in diploblastic embryos (2 germ layers, e.g., Cnidaria: *Nematostella vectensis* and stony corals), we needed a model that was able to simulate mechanical properties that can create shape changes in individual cells that lead to invagination. The cells in such a model should be individual entities that can move freely, can have different properties on the cell (heterogeneity), and deform naturally, capturing detailed cell shapes and interactions, and complex tissue dynamics, with more realistic results. Forces should be able to be transferred between cells and propagate in all directions, and the effect on the total final shape of the embryo should be able to show asymmetries and spatial variations [35]. Deformable cells can be modeled with Lattice-based models, such as the Cellular Potts Model (CPM) [36]. These models represent space with a grid, simplifying computational processes but limiting their ability to transfer forces and capture complex biological shapes and deformations naturally. This makes them less suitable for studying dynamic processes like apical constriction and adhesion during embryonic development. Off-Lattice deformable cell models on the other hand, allow for capturing a more natural shape. For example, vertex models [12,13,17,18,27,37] and multi-particle models (SEM [38]). In the multi-particle models, the particles that form one cell in this type of model, interact with each other via potentials and allow for capturing cellular deformations in more detail. The connections between these particles are dynamic i.e. they can move independently and migrate to other positions within the simulated cell. This make it difficult to assign regions with specific cellular properties. In vertex models, vertices are interconnected by edges, forming a polygon (2D) or polyhedron (3D), which allows for assigning regional properties and are able to model cellular mechanics (force transfer) more accurately [39] compared to particle based models. The hollow structure of polyhedrons also reduces computational demands compared to multi-particle based systems, but now an internal pressure has to be modeled to conserve cellular volume. Several 2D vertex models already exist that have studied cell sheet bending in for example, *Nematostella vectensis* and *Drosophila* embryos [12,13,17,18,27] and the mechanical properties that lead to it. These 2D models, that have a good likening to in vivo results [12], show that apical constriction together with cell adhesion, seem to be the main driver of cell sheet bending. They also demonstrate that shape changes can be a passive response to the active deformations in the system [12]. However, 2D models, although elegant in their simplicity, do have some drawbacks. They lack the interaction with neighboring cells in the third dimension and make the assumption that a spherical (radially symmetrical) or, in the case of *Drosophila*, oval embryo, can be modeled using a 2D cross section through the middle of an embryo. This seems logical, but automatically excludes most of the embryo that falls outside this plane. Anterior or posterior shape effects, or asymmetrical properties that arise during gastrulation in the embryo, will be missed. In real embryos, forces propagate in all directions (hoop stress), distributing the force in every dimension. However, the forces generated by a virtual cell in 2D can only travel in 2 dimensions, making them more pronounced than in reality would be the case. The lack of hoop stress in 2D simulations creates shape deformations that in reality do not seem to occur [12]. In a 3D embryo, the neighboring cells (that are not modeled in a 2D simulation) resist the shape changing forces created by the infolding process. Although, vertex models offer a more accurate representation of cell behavior, models with shared vertices between adjacent cells prevent independent cell movement [13,17,18,27,37], making these types of models unsuitable for our study. Based on the above, we argue that an inherently 3D process like embryogenesis can be better modeled with a 3D vertex model with separate cells. We therefore developed our own 3D deformable cell based model, where cells are represented by a detailed polygon that has a conserved cell volume, and definable regions with different properties (stiffness, adhesion, constriction). This allows for heterogeneity of mechanical properties on the single cell. When multiple adhesive cells are simulated close to each other, they can form a larger structure. The properties of all individual cells together determine the emerging behaviors of the super structure.

Our model uses an internal scripting language to set up the simulation experiments. Here, we can set the properties of each individual cell, making it much less labor intensive to design and run simulations. The genetical and biochemical properties are modeled implicitly by the chosen properties and parameters.

This model was used to qualitatively study the cellular mechanical properties (apical constriction, cell-cell adhesion, and cytoskeletal stiffness) and their effect on cell shape changes that lead to invagination in diploblastic embryos. Although a model like this is computationally heavy, it will better capture the biological circumstances that influence morphogenesis, and thereby help to answer the question: which mechanical cell based mechanisms are necessary for cell sheet bending in 3D, and how does this compare to 2D simulations? Using a 3D model also allowed us to test the effect of geometrical properties like endodermal plate shape, the number of cells in a blastula, number of endodermal cells, and the start position of constriction in the endodermal plate (constriction mode), on the global embryo shape. We qualitatively validated the model by comparing cell shapes and embryo shapes to existing 2D models and biological data of *Nematostella vectensis*, Stony corals, and *Drosophila*, which was readily available [10,11,17,33,40–46]. For this we created planar simulations (3D cells placed on a circle in a 2D plane) (see S2 Appendix), representing a cross section through a blastula. These planar simulations also allowed us to test the model properties.

### Model.

**Fig 3:**
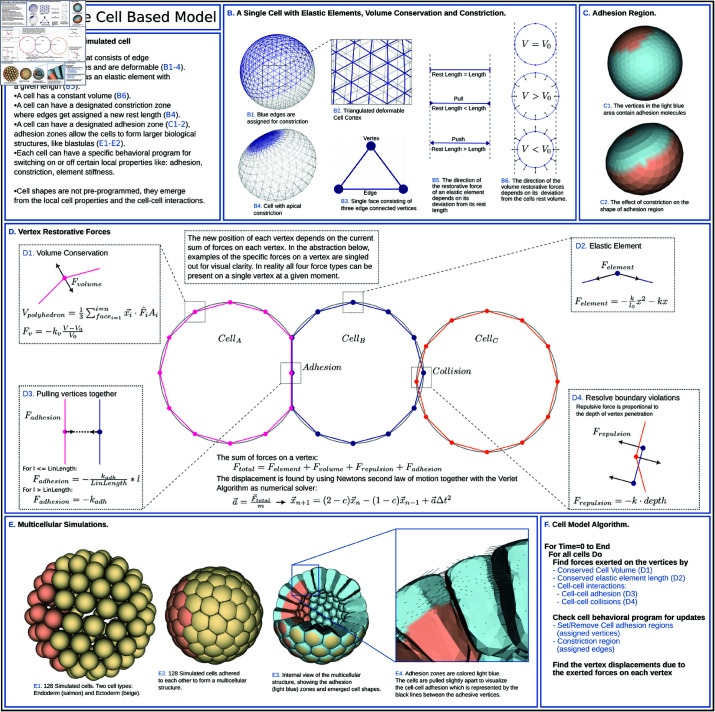
3D Deformable cell based model. This figure introduces the 3D deformable cell based model. The model simulates a single cell, which is a distinct entity with its own local properties that can interact with other cells to form a larger structure. Each 3D cell is formed by a detailed polyhedron surrounding a conserved volume, making it freely deformable and movable. The boundary of this polyhedron consists of a triangulated mesh representing the biological cell cortex, composed of interconnected vertices and elastic elements that adapt to applied forces. The cortex can actively deform by constricting edges or passively by interaction with neighboring cells. The vertices and edges allow for different regions to be placed on the same cell to simulate cellular properties like adhesion, stiffness, and constriction of the cortex. These properties can change over time according to predefined rules encoded in a basic scripting language executed by the model code, effectively simulating genetic traits that govern cellular behavior. Multiple of such cells can be simulated in the same space to form larger entities or structures, such as blastulas. The cells can be pushed when they are adjacent but unconnected to other cells, or be pushed and pulled when they are adhered to other cells. Panels (**A–F**) explain the cell model concept and how multiple cells together can simulate a hollow blastula, where cells adopt a wedge shape due to the local cell-cell interactions rather than by pre-programming the shape. (A) Description of the properties of the simulated cell. (B) A single cell with: *Elastic elements*, *Volume conservation* and *Constriction*. Images (B1, B4) show a single cell that has a deformable cortex. Images (B2–B3) illustrate the cell cortex that consists of edge interconnected vertices. Image (B5) depicts the edges that are modeled as a restorative *Elastic element* with a given rest length. When the actual length deviates from the rest length, a restorative force is generated (push or pull). Image (B6) illustrates *Volume conservation*. Deviation from the cells rest volume results in a volume restorative force. In image (B1), the dark blue edges in the apical, top of the cell, region (0–50%) of the spherical cell (with uniform cell stiffness) are assigned to constrict. Image (B4) shows the cell after the edges have constricted. The constricted apical edges have reduced their length, resulting in a flattened apical area and expanded lateral-basal region due to the volume conservation. The cell shape still resembles the initial spherical shape. (C) Adhesion region. Image (C1) shows the adhesion region on a single cell, visualized in light blue, where the vertices within this region are adhesive. Image (C2) depicts the same cell but now the edges in the apical region have constricted (see also Image B4). The adhesion region has now moved apically, reducing the area of the adhesion region, but not the number of vertices. This also results in the expansion of the basal non-adhesive region. (D). Vertex Restorative Forces. This panel is an abstraction of the model, to highlight the different forces that can work on a single vertex: (D1) *Volume Force*, (D2) *Elastic Element Force*, (D3) *Adhesion pulling Forces*, and (D4) *Collision Forces* that resolve boundary violations. These forces together result in *F*_*total*_, that determines the new position of a vertex (See also Methods and S1 Appendix). (E) Multi-cellular simulations. Multiple cells can be adhered together to form a larger structure, here they form a hollow blastula. Image (E1) shows 128 single cells that are assigned a cell type, endoderm (salmon) and ectoderm (beige). Image (E2) demonstrates cells that are adhered together to form a blastula. The cells here have a stiff apical (outer) area, an intermediate stiff lateral region, and a soft basal (inner) area. This pulls the apical area flat and extends the basal area, changing the cell shape from round to elongated wedge shaped. Opening up the blastula in Image (E3) shows the emerged cell shapes and the two different sizes of the adhesion regions (light blue) placed on the two different groups of cells (endoderm and ectoderm). The endoderm has a small apical band (light blue and salmon), while the ectoderm is fully adhesive (completely light blue). Image (E4) features a close up of the cells to visualize the adhesive bonds (dark blue) between the vertices in the light blue zones. The cells are pulled apart slightly to stretch the adhesive bonds, making them more visible. (**F**) Cell model algorithm, shown as pseudo code.

Our model builds on classical vertex models that have been used to study tissues during development [47,48]. Internal forces and external constraints determine geometrical shapes in such models, which is why they have been useful to study cell shapes and tissue morphogenesis. Individual 3D cells are made of a mesh of vertices that form a polyhedron (Fig 3B 1-3). To simulate polarity, the cell can be divided into regions (see Methods). These regions can have biological cellular properties like cell stiffness, cell constriction and cell-cell adhesion. Different cells can have different properties. Our model is based on Newtonian mechanics. The force generating circumstances are visualized in Fig 3 panel D in a simplified manner (see also Methods and S1 Appendix). Here, we highlight where the restorative forces (*Volume conservative force*, *Elastic element*, *Adhesive force*, and *Collision detection*) are acting on the system and how the forces are calculated. Together (*F*_*total*_) they determine the next position of the vertex, which is found by using the *Verlet algorithm* (see Methods and S1 Appendix). The effect of the *Volume conservative force* (Fig 3D 1) on the cell is depicted in Fig 3B 6. The working of the *Elastic element* (Fig 3D 2) on a cell is shown in Fig 3B 5, where an edge has a rest length that the element tries to preserve. Apical constriction, which is essential for gastrulation, is implemented by decreasing the rest length of the elastic elements that connect the vertices, to a chosen value (constriction factor). The effect of apical constriction on a single cell, is demonstrated in Fig 3B 4 and 3C 2. Cell-cell adhesion, which is crucial for developmental processes, is achieved by making individual vertices in a region of the cell adhesive (Fig 3C 1). As a consequence, two cells next to each other, within a given distance, will experience an *Adhesive force* (Fig 3D 3) and will stick together. The distance (*l*) between the cells at this points is pulled to *l* = 0 (see Methods). Adhesion is crucial for the purpose of simulating an embryo (see Fig 3E 2-4). The modeled cell is the central unit in our system. Multiple cells can be simulated in the same space. When forming structures with multiple cells, like blastulas, *Collision detection* (see Methods) comes into play, resolving boundary violations. The repulsive forces are placed on the vertex that has violated the boundary and the triangle that has been penetrated, moving the vertex out, causing local deformations. This causes the cells that form a blastula to deform freely, depending on the constraints from neighboring cells and volume conservation (Fig 3E 2-4). Adhesion and collision are costly operations, that have been optimized using Axis Aligned Bounding Box placed around cells (see Methods). This resulted in a linear relation between the number of cells and the execution time, allowing us to run the model on a cpu system.

Taken together, the biologically inspired properties of our 3D vertex model: the elastic cortex, constriction and cell-cell adhesion, allow us to perform 3D simulations of early development with detailed embryonic shape changes.

## Results

Our vertex model was specifically developed to simulate the bending of a cell sheet (invagination) in a blastula. Using a planar configuration (3D cells in a plane, see S2 Appendix) we explored the model properties and validated that we could qualitatively reproduce previous results from Odell *et al*. [1981] and Tamulonis *et al*. [2011][12,13]. Next, we applied this knowledge to run the different 3D simulations that follow below.

### Constriction.

**Fig 4:**
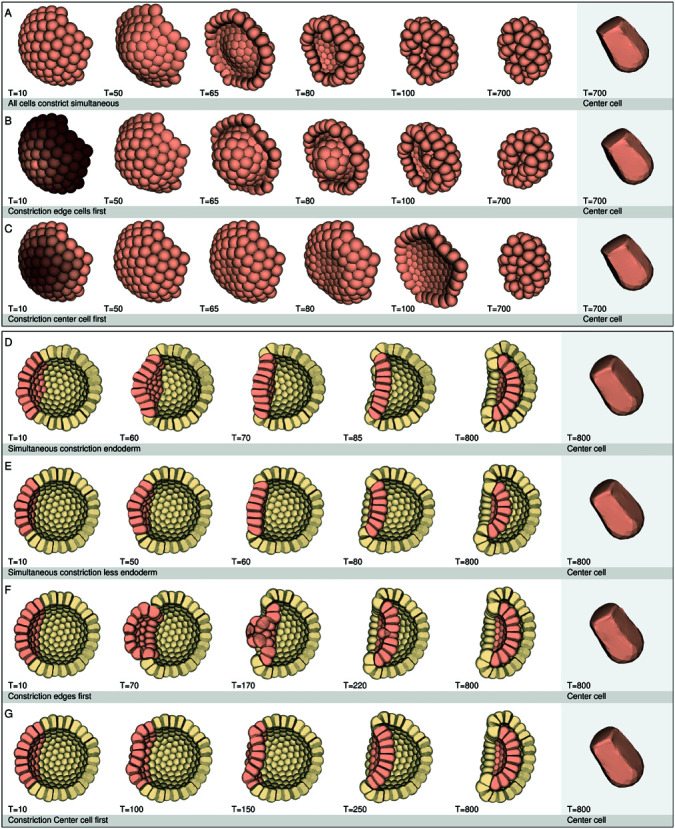
Constriction modes. The images show time series of detached endodermal plates and whole blastulas with different constriction modes. The adhesion regions are located apico-lateral (20-65%), the constriction factor is 0.1, and the cell stiffness is uniform in all cells (k = 0.5). (**A**– **C**) Detached endodermal plates with 83 cells. (**D**– **G**) Blastulas with 256 cells. The endoderm cells are colored salmon. (A) Simultaneous constriction of all cells. (B) Edge cells (brown gradient) constrict first, with time interval between constricting cells, 100 units. (C) Center cell (brown gradient) constricts first, with time interval between constricting cells, 100 units. (D) Simultaneous constriction of all the endoderm cells (58) in a blastula. (E) Simultaneous constriction of all the endodermal cells (36) in a blastula. (F) Ring constriction of the endodermal cells (58) in a blastula. Edge cells constrict first. (G) Ring constriction of the endodermal cells (58) in a blastula. Center cell constricts first. Simultaneous constriction in a detached endodermal plate (A) caused the edge cells to curl up first. Because all cells constricted simultaneously, the center of the plate flattened faster, than when the edge cells constricted first (B). Constricting the center cell first (C), created a dip in the center of the plate before the edge cells curled up. Constricting endodermal plates in an embryo showed similar results as with a detached endodermal plate. When cells constricted simultaneously (D), this caused the edge cells of the plate to move inwards first, which pushed the center of the endodermal plate outwards. With fewer endodermal cells (E), this effect was less pronounced and the plate became flatter sooner before invaginating. When the edge cells constricted first (F), the top of the endodermal plate formed an extreme bulge, where the cells were almost pushed out before invaginating in. Constricting the center cell first (G) and continuing ring by ring created a concave shape in the center of the plate right at the beginning of invagination. These simulation experiments show that the endodermal plate shape during the invagination process reveals something about the timing and the constriction mechanism in the endodermal plate.

Constricting a single detached cell (Fig 3B 4), shows that without geometrical constraints in the form of other cells, apical constriction alone was not enough for cell elongation to occur. Therefore, multiple cells were adhered together to form a plate (with 83 cells) and a blastula (consisting of 256 cells). These plate and blastula were used to test the effect constriction has on the local cell shape and the global plate and embryo shape. For this, the endodermal plate was divided into rings, starting with a single cell in the center of the plate followed by concentric rings (referred to as ring constriction, Fig 4B and 4C, brown gradient). We examined three different constriction modes: Mechanism 1). All plate cells constrict simultaneously; Mechanism 2). Ring constriction starts at the edges of the plate, with a time interval between constricting rings; Mechanism 3). Ring constriction starts at the center of the plate, with a time interval between constricting rings.

Fig 4A–4C shows the results for the detached plate simulations (time series). The adhesion region in these plates is 20-65% of the spherical cell. The cells for mechanism 2 and 3 constrict with a time interval of 100 time units between cells. S1 Fig shows again plates with an adhesion region of 20-65% of the spherical cell, but now with a time interval of 500 time units for mechanism 2 and 3. S2 Fig shows plates with an adhesion region of 20-100%, with a constriction interval of 100 time units. These images show that the plates where the cells constricted simultaneously (Fig 4A), curled up at the edges before the center of the plate was leveled out, even though constriction was simultaneous for all cells. The plates where the edges constricted first (Fig 4B), seemed to curl up in a similar manner, except now the center of the plate remained convex much longer before it became concave. Plates where the center constricted first (Fig 4C) showed a dip at the center of the plate, before the plate flattened out and the edges curled up. Increasing the time interval (to 500 time units) between constricting cells (S1 Fig) showed a more pronounced bulge when the edge cells constricted first, and a more pronounced dip when the center cell constricted first. The end results of the plates were more or less the same for all experiments, forming a sort of closed oval shape. Plates where the cells were adhered 20-100% (S2 Fig) showed similar results to the 20-65% plates (Fig 4A-4C) with time interval 100 time units. The images of the single cells in Fig 4A-4C (right images) show the center cell of each of the plates. These cells are elongated compared to the single cell experiment (Fig 3B 4). Cells of plates with 100% adhesion (S2 Fig) that constricted simultaneously or the edges first, due to the full adhesion, had a slightly wider basal area.

We repeated the endodermal plate experiments with different constriction modes, but now in an embryo of 256 cells. The endodermal cells are colored salmon. Constricting all endodermal cells simultaneously (no time interval between constricting cells) (Fig 4D) caused first an inward movement of the edge of the endodermal plate attached to the ectodermal cells and an outward movement of the top of the endodermal plate (convex shape), similar to the detached plate simulation (Fig 4A). The plate then flattened out, before it moved inwards. The gastrula shape became bowl-like with a wide blastoporal opening. Time point T=85 resembles stony coral invagination (Fig 2J). Again, constricting all endodermal cells in a blastula, but now with fewer endodermal cells (Fig 4E), shows a less pronounced outward bulge of the center of the endodermal plate. When the edges of the endodermal plate constricted first (Fig 4F), a stronger outward bulge was created than with the simultaneous constriction mode, almost pushing the final constricting cells out of the plate before finally invaginating. A blastula where the endodermal plate started ring constriction at the center (Fig 4G), got a dip in the center of the plate that continued to expand towards the edges of the plate during invagination. This immediately created a concave invagination in the plate from the beginning. We find that the different constriction modes translate into different plate shapes during the invagination process, even though the end results are qualitatively similar.

### Cell stiffness and constriction factor.

**Fig 5:**
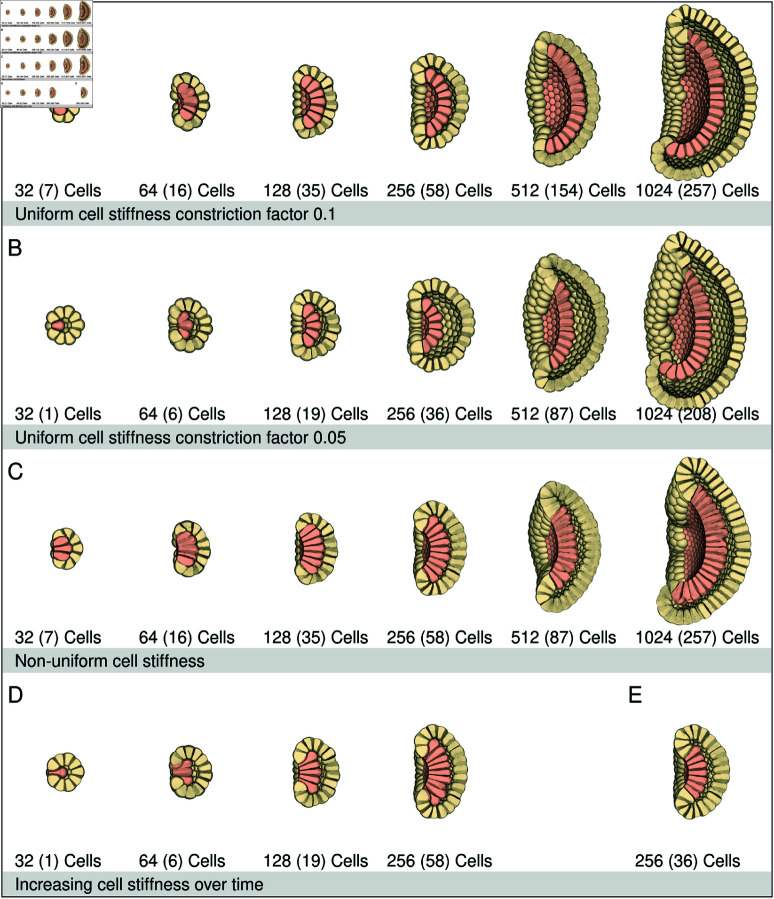
Cell Stiffness and constriction factor. This figure shows cross sections through blastulas that have constricted the apices of the endodermal plate. The endodermal plate (salmon) is oriented to the left. Below each blastula is the total number of cells in the blastula (32-1024) and number of endodermal cells between parentheses (). (**A**) Uniform cell stiffness constriction factor 0.1. Simulating with the following parameters: Cell stiffness 0-100% k=0.5. Constriction factor 0.1 and endodermal adhesion region 20-65%. All blastulas invaginated, but the global shape was bowl-like with a large opening. In the 1024 blastula the endodermal and ectodermal layer did not establish contact. (**B**) Uniform stiffness with constriction factor 0.05. Simulating with the following parameters: cell stiffness 0-100% k = 0.5, constriction factor 0.05, endodermal adhesion region 20–65%, The endodermal cells elongated and expanded more basally due to the smaller edge constriction factor. This placed more strain on the smaller embryos, therefore the number of endodermal cells were reduced to allow better invagination into the blastulas. Reduction of endodermal cells strongly influenced the global shape. The final gastrula was rounder than seen in Fig 5A but now no germ layer alignment occurred. All blastulas invaginated, but the larger the embryo size, the more bowl shaped the embryo became with a larger opening. (**C**) Non-uniform cell stiffness. Simulating with the following parameters; constriction factor 0.05, endodermal adhesion region 20–65%. The cells are divided into three stiffness zones (apical region: 0-30% of the spherical cell, lateral region: 30–70% and basal region: 70–100%). The cell stiffness is stiffest in the apical region (*k* = 1) in the lateral region the stiffness is *k* = 0.5 and the basal region is the softest (*k* = 0.1). All the blastulas invaginated and the endoderm and ectoderm aligned completely in the smaller gastrulas. The larger gastrulas (512 en 1024 cells) approached alignment. The global shape became bowl-like, with a large opening. (**D**) Increased cell stiffness over time. The endodermal cell stiffness is increased apically and decreased basally over time. Simulating with the following parameters: Constriction factor 0.1, endodermal adhesion region 20-65%. The cell stiffness at the start of the experiment is *k* = 0.5. For the endoderm cells the stiffness of the apical top (0–40%) is slowly increased to *k* = 1.5 and the basal side (40–100%) decreased to *k* = 0.1. For the ectoderm cells the apical stiffness is increased to *k* = 1.4 and for the basal side it is decreased to *k* = 0.35. The cells showed a more bottle like appearance close to the ectoderm (blastoporal lip cells). The layers did not connect. The smaller gastrulas remained rounder, but the largest gastrula (256 cells) had a strong bowl-shape and large opening. (**E**) Number of endoderm. Image E shows the 256 celled blastula from row C, but now the number of endodermal cells is reduced from 58 to 36. This results in more blastocoel space remaining between the endodermal plate and the ectodermal layer. The smaller number of endodermal cells resulted in one more ectodermal ring that could move into the blastoporal opening.

In nature, depending on the species, blastulas that are ready to gastrulate can consist of different cell numbers (blastula sizes). In *Nematostella vectensis* embryos, the blastula with blastocoel is formed at the 32-64 cell stage. Therefore, the smallest blastula cell number used here is 32. The maximum cell number is 1024 (2^10^, 10 rounds of cell cleavages), which is when *Aurelia aurita* embryos start to gastrulate. These blastula sizes (32-1024) were used to determine the morphological effects different cell numbers, stiffnesses and constriction factors have on invagination in a 3D simulated embryo. The number of endodermal cells of a blastula is determined by the rings and the cells therein, that form the endodermal plate. The endoderm cells are only adhered together apico-laterally (20-65% of the spherical cell). The ectoderm of the blastulas is fully adhered together (100%). The adhesion keeps the cells closely attached. Fig 5A shows gastrulas with uniform cell stiffnesses (Parameter values are given in S1 Table). Due to the apical constriction factor (0.1), the apical area became smaller, and the endodermal cells became more elongated, causing cell sheet bending. The embryo shapes became bowl-like. In the 1024 celled gastrula the germ layers did not align (see S1 Video). With a smaller edge constriction factor (0.05 instead of 0.1), cells elongated more (Fig 5B), and fewer endoderm cells could move into the blastocoel in the smaller embryos. Therefore, the number of endoderm cells was reduced in all blastulas. This influenced the global shape of the gastrulas, which remained rounder compared to the results shown in Fig 5A, since fewer cells invaginated, and more cells remained ectoderm. However, now no germ layer alignment occurred in any of the gastrulas.

Fig 5C shows gastrulas with non-uniform cell stiffnesses. The cell surface was divided into three regions: apical (0-30%), cells stiffness k=1, lateral (30-70%), cell stiffness k=0.5, and basal (70-100%), cell stiffness k=0.1. These gastrulas have longer endodermal and ectodermal cells compared to the uniform cell stiffness gastrulas (Fig 5A and 5B). Some endodermal cells start to differentiate their cell shape, resembling bottle-like cell shapes [12]. The stiffer apical area caused the outer surface of the embryo to become flatter. Under these conditions, only the 512 and 1024 celled gastrulas did not align the germ layers (Fig 5C). When the number of endoderm cells of the 256 cell blastula with non-uniform cell stiffnesses were reduced from 58 to 36 (Fig 5E), more ectoderm moved into the blastoporal opening, but now the endoderm did not fully align with the ectodermal layer. Fig 5D shows simulation results where the stiffness of the apico-lateral region (0-40%) of the endodermal cells was increased over time from k=0.5 to k=1.5. The basal-lateral region (40-100%) had a stiffness of k=0.1. The ectodermal apico-lateral cell stiffness was increased from k=0.5 to k=1.4 and the basal-lateral cell stiffness was k=0.35. The gastrulas show bottle shaped endodermal cells. These cells have a narrow elongated apical neck with a broader basal body. The cells with the most profound bottle shapes are found close to the ectodermal edge.

The shape of a cell depends on its position in the embryo. In S3 Fig the center aboral ectodermal cell, the center endodermal cell and a blastoporal lip cell of the 256 celled gastrula of Fig 5C, are visualized and compared to a spherical cell. The position of the cells has, apart from the different shapes, also an effect on the cell surface area. The cell with the largest surface area was found in the blastoporal lip.

### Apical shape.

In *Xenopus leavis* embryos, the constricting cells form a ring on the embryo with stretched apices. In isolated explants, these same cells have spherical apices [4].

**Fig 6:**
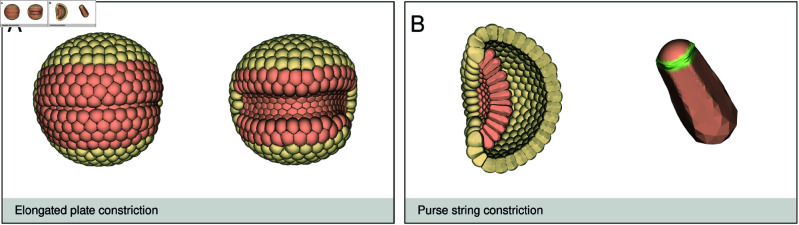
Apical shape. (**A**) Linear row of endodermal cells. This image shows two time points of an embryo with 512 cells that has an elongated endodermal plate shape. The simulation parameters are given in S2 Table. Constricting one row of endodermal cells mostly reduced the apical area of the cells vertically, due to the deformability of the passive surrounding cells and resistance of the constricting cells. When more rows constricted, the apical areas became rounder again, since now the force pattern changed (see S1 Graph). (**B**) Endodermal purse string constriction. The endodermal cells of a 512 celled blastulas with 87 endodermal cells constricted using a purse-string method. The single cell shows the center cell from this gastrula with a purse string constriction. The green zone indicates the constriction region. Due to the outward volume pressure created by the purse string constriction in the cell, the apical top stiffness also had to increase. During constriction the cell stiffness of the apical top (0–20%) was increased from *k* = 0.9 to *k* = 1.8 and the 20–60% zone from *k* = 0.5 to *k* = 1.4, to prevent excessive bulging of the apical area.

The previous results, Fig 5, and S3 Fig (cell area) have already shown that the geometrical setting of cells determines how they are shaped. Here, we show how the apical area is influenced by the geometrical setting. Fig 6A shows a spherical embryo with a rectangular shaped plate of cells (colored salmon). The center row in the plate has constricted. The cells apical area in this single constricted row has reduced vertically, but barely horizontally. When the rows above and below the center row are constricted, the apical area of these rows remains larger and the area of the first row is increased again. The graphs in S1 Graph shows the vertical and horizontal length of the apical area of the cells in the endodermal plate over time. The graphs show that the rows 1-4 above and below the center row are stretched vertically before they started constriction. Every extra row that constricts pulls on the rows that constricted before them, stretching the apical area again. This is visible in the wave, or pulsed pattern of the vertical graph. The last row to constrict, can constrict its apical area the most vertically since the adjacent ectodermal cells are flexible and do not oppose the constriction forces as much as the constricted cells do. Horizontally the center row can initially constrict the least, because the cells are inhibited by the surrounding cells, but it can eventually slowly constrict further and decrease its area. The horizontal apical area of most cells appear to be in the same range (0.7-0.9).

The previous constriction mechanisms were by flat apical constriction. However, Magie *et al*. [2007] [11] suggest that in *Nematostella vectensis* apical constriction is by a purse string mechanism, where the apical region, contrary to *Drosophila* and *Clytia hemisphaerica*, does not flatten, but bulges outward. Fig 6B shows purse-string constriction. In the single cell (right image), the green area indicates the constricted region. Instead of apical flattening, we now saw a small outward bulge at the apical region of a cell. An increased apical stiffness was necessary to prevent the apical surface from bulging outwards too much. The overall shape of the plate and the global embryo shape did not differ much from flat apical constriction.

### The endodermal plate and blastoporal opening.

**Fig 7:**
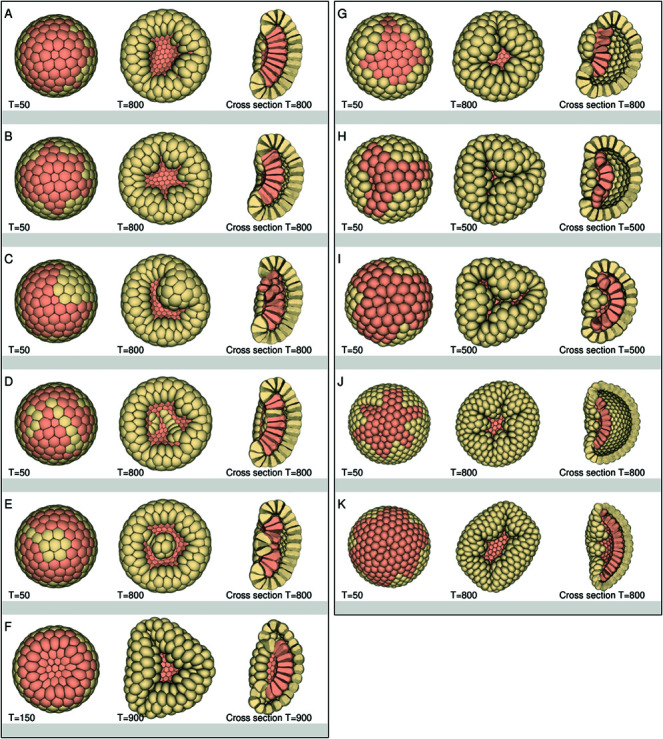
The endodermal plate shape and blastoporal opening. This figure shows different blastulas (256–512 cells) with different endodermal plate shapes. The front view shows the oral side with the initial (T = 50) and final endodermal plate shape, and the cross section at the end of the simulation. The numbers of endodermal cells depends on the plate shape; Plate shape: (A) 67 cells, (B) 64 cells, (C) 69 cells, (D) 71 cells, (E) 73 cells, (F) 58 cells, (G) 31 cells, (H) 32 cells, (I) 64 cells, (J) 64 cells, (K) 128 cells. The parameters of each blastula are given in tables S2 Table and S3 Table. In all simulations the plates of the blastulas invaginated regardless of the shape. However, not all endodermal plates connected with the ectodermal layer. All the gastrula shapes became bowl-like. The blastula in row (F), had a spherical plate with softer ectoderm and smaller adhesion region between the ectodermal cells, which helped to close the blastoporal opening more. The oral view shows an angular shape and folds in the embryo. The plate shapes in row (G), (H) and (I) allowed for a better closure of the blastoporal opening, but not a better layer alignment. Only gastrula I seemed to align the layers better, except for the blastoporal lip region, where the ectoderm interrupted the endodermal plate. Row (J) and (K) show blastulas with 512 cells in a star shaped pattern. Row (J) had a better closure of the blastoporal opening, but the layers did not connect. While the layers in row (K) (larger star shape plate) did connect, but now the opening remained large.

Up to now we assumed that an endodermal plate would be spherical, like those found in Stony corals [9], but in *Nematostella vectensis* endodermal plates do not necessarily have to be spherical [10]. We created different plate shapes (see Fig 7A–7E and S2 Video) and tested how robust the invagination process is when an endodermal plate is not spherical in shape. The figures show that irregular shaped plates could still invaginate and reach the aboral side, but the embryos shape stayed bowl-like with a large blastoporal opening.

In *Nematostella vectensis*, *Aurelia aurita*, and Stony corals [9,11,32] the future blastoporal opening immediately becomes smaller again after invagination and closes, forming spherical embryos. The 3D invagination simulations (Figs 5A–5E and 7A–7E) show that the global embryo shapes were bowl-like and the blastoporal opening did not close but remained wide, opposed to the planar simulations (See S2 Appendix), where the opening often became smaller during invagination and the embryos became rounder again. We explored two different mechanisms to study how the blastoporal opening could be closed and how this affected the embryo shape. First, we used softer ectoderm, which allowed the ectoderm cells to stretch and reshape, closing the blastoporal opening more (Fig 7F). This, however, had no effect on the global shape of the embryo, which remained bowl-like.

Next, we focused on the shape of the plate. For this we created plate shapes with a triangle, T-shape and star shape in 256 and 512 celled blastulas (Fig 7G–7K). These shapes helped remove folds in the blastoporal region that would otherwise prevent blastoporal closure, but now the gastrulas did not always align the germ layers and the embryo shape remained bowl-like.

## Discussion

To study the driving forces behind invagination, we looked at existing data of model organisms, such as *Nematostella vectensis* (sea anemone), *Aurelia aurita* (common jellyfish), Stony corals (*Platygyra contorta, Favites abdita, Dipsastraea (Favia) speciosa*), (which are all Cnidaria), and *Drosophila melanogaster* (fruit fly) [9–11,32,43,45,49–51], that all use invagination to bend a cell sheet inwards. However, identifying the exact processes that can cause invagination in vivo can be difficult due to many interacting cellular processes. Therefore, 2D simulation models have been used to study invagination [12–15,17,22,27], which have shown that apical constriction and adhesion are important properties to create cell shape changes that can bend the endodermal plate into the blastocoel during invagination [26]. These 2D models, however, lack the ability to implement geometrical properties and asymmetries found in real natural 3D blastulas and show the role they play in the eventual global shape an organism adopts.

In this study, we developed a 3D deformable cell based simulation model with a Newtonian force based approach. Our choice of making a 3D vertex model was driven by the need to accurately capture shapes of individual cells in detail and the complex mechanics of embryonic development. The ability to model separate cells with regional heterogeneity, efficiently simulate physically accurate force transfer, and represent continuous deformation makes this model well-suited for studying how mechanical forces reshape tissues over time. It provides a powerful framework for studying the impact of mechanical properties on complex cellular behaviors and understanding morphogenetic processes in 3D systems. It also offers insights into how differences in local properties can lead to large-scale tissue changes without pre-programmed patterns and enabled us to qualitatively compare simulation results with real biological tissue shapes. Although, we here modeled invagination in a diploblastic organism, the cell model is not restricted to modeling a hollow blastula. Due to its modular concept, the model could be used to form any biological entity to study the effect of local cell properties on tissues as a whole and the behavior of these tissues in different biological contexts, when cross-sections do not capture the true nature of the system.

We validated and tested the cell based model by performing planar simulations (3D cells in a 2D plane). The outcomes of the simulations were qualitatively compared both to available real biological data from the literature and true 2D simulation results [12,13]. We observed that with the planar simulations, we found shapes that resembled not only the biological data, but also the results from existing 2D models [12,13], both cellular as well as embryo shape.

### The effect of constriction mode.

During the invagination process in an embryo, the cell sheet undergoes shape transitions while bending inwards, which can differ in different species (e.g. *Nematostella vectensis*, *Aurelia aurita*, *Dipsastraea (Favia) speciosa* [9,31,32]. The invaginated plate shape observed in *Aurelia aurita* embryos (Fig 2K) are concave. However, the initial invagination shape of endodermal plates in *Nematostella vectensis*, *Favites abdita* and *Dipsastraea speciosa*(Fig 2A, 2G and 2J) are less concave than the plates shapes found in *Aurelia aurita*. Assuming that the basic mechanisms for invagination are shared between species, then these different invagination shapes could indicate that there are variations in how the mechanisms (constriction modes) contribute to the infolding process across different species. We tested how different constriction modes (constricting all cells simultaneously, or start constriction at the edge of the plate, or start constriction at the center of the plate) in detached plates and 3D embryos affected the tissue shape. These simulations not only showed that cell-cell adhesion and apical constriction are enough to bend an endodermal plate, but also that the mode of constriction, and constriction time interval, influenced the shape transitions seen during the constriction process. Simultaneous constriction resulted in that the edge cells reduced their apical area first. In the detached plate this was seen by the curling up of the edges, while in the simulated embryo the plate buckled downwards at the circumferential edge and upwards in the center before it flattened and became concave. This can be explained by the fact that in a single detached cell (Fig 3B 4) the cell tries to reduce its apical area and push its volume basally. This results in lateral expansion due to the elastic properties of the cell cortex. However, in an endodermal plate or blastula, adhered cells form a geometrical constraint that opposes these forces and pushes the volume of the constricting cells basally, thereby elongating them. During simultaneous apical constriction, all the cells try to constrict their apices at the same time, thereby resisting apical deformation due to the stiffening of the apex during constriction. The ectodermal cells, however, do not constrict and are thus apically more flexible than the endodermal cells. In the detached plate there are no surrounding edge cells to delay deformation. The edge of the endodermal plate is therefore the first region that can move as a response to the forces that are generated by constriction, even though the timing of constriction is simultaneous in all cells. After that, a wave moves through the plate, to the center, of cells reducing its apical area. This flattens the plate before making it concave, finally causing invagination into the blastocoel. The flat plate shape seen in stony coral embryos [9] (Fig 2J), resembles the plate shape seen during simultaneous constriction, suggesting that the time interval between the constricting individual cells is close together in this organism.

Asynchronous constriction (with time intervals between predefined constricting groups of cells) that started at the center of the plate, caused a dip at the constriction site, that expanded when more cells constricted concentrically, resembling the plate shape seen in *Nematostella vectensis* (Fig 2A-2D) and *Aurelia aurita* (Fig 2K). When cells at the edge of the plate constricted first an extreme bulge emerged at the center of the plate. The mechanism of how a plate constricted, influenced its appearance during the invagination process, even though the end states were visually similar. The constriction mode (synchronous versus asynchronous, and center or edge start position) therefore determines the shape the plate adopts during invagination and gives us insight into how the invagination process can take place in different species.

### Geometrical setting.

The effect of the geometrical surroundings on the cell shape in our 3D simulations, is shown by constricting a single row of cells in the center of a rectangular plate in a spherical embryo. The apically constricted cell areas reduced vertically, but not as much horizontally, creating a horizontally elongated area, even though the constriction force on the apical area was isotropic. This horizontally elongated apical shape is caused by the passive expansion of the apical area of neighboring cells above and below the constricting cell row, while the constricting cells in the center row resist deformations in the horizontal direction. When neighboring rows above and below the center row constricted, the vertical pulling force from these neighboring cells pulled the apical area of the center row more spherical again. By putting a uniform apical constriction force on the individual cells, in an elongated plate (asymmetrical situation), the constricting cells autonomously reshaped, creating an anisotropic shape pattern. These results resemble the model results of Fierling *et al*. [19], where they put isotropic stress, in the form of constriction, on the mesodermal cells of an oval *Drosophila* embryo, which resulted in an anisotropic shape pattern in the furrow.

In contrast, the embryos of Fig 5, which have the same uniform constriction force in a spherical (symmetrical) setting, created a spherical (isotropic) shape pattern. This shows, that in the model, the mechanical properties determine how a cell can respond to the forces created by neighboring cells that act upon them, but the geometrical setting determines the eventual shape the cell and apical area can become. Cell shape therefore does not necessarily have to be genetically predetermined [52].

The constriction region determines if the apical shape becomes flat or stays convex. Full apical constriction can create a flat apical area (e.g., *Drosophila melanogaster*, *C. elegans* [11] and *Clytia hemispaerica* [28]), while a purse string region only constricts the region just below the apical area, creating a convex apex (e.g., *Nematostella vectensis* [10–12]). We found that the type of constriction did not affect the invagination process, only the appearance of the local cell shape and the embryo; the surface of the endodermal plate was less smooth with purse string constricting cells, than with flattened apical constricting cells. However, when a purse-string mechanism was used, the apical area needed to be stiffer to provide a counter force to the volume pressure, otherwise the apex ballooned outwards.

### 3D Geometrical properties.

We have shown that the shape cells adopt is a consequence of their mechanical properties, their surroundings and how they interact with it. The cells can actively change their shape and through adhesion, affect both their own shape as well as their neighbors, which influences the progress of the infolding process and the shape of the embryo. The embryo shape, however, is also influenced by geometrical properties that are present in the pre-gastrula, like the plate shape. Changing the endodermal plate shape from spherical to irregular in hollow blastulas showed that the plate shape does not really matter for the bending of the plate and invagination process. As long as the constricting cells in the endodermal plate were adhered together, the embryo invaginated, which shows that invagination is a very robust process that can resolve the natural variation that is present between embryos [53].

**Fig 8:**
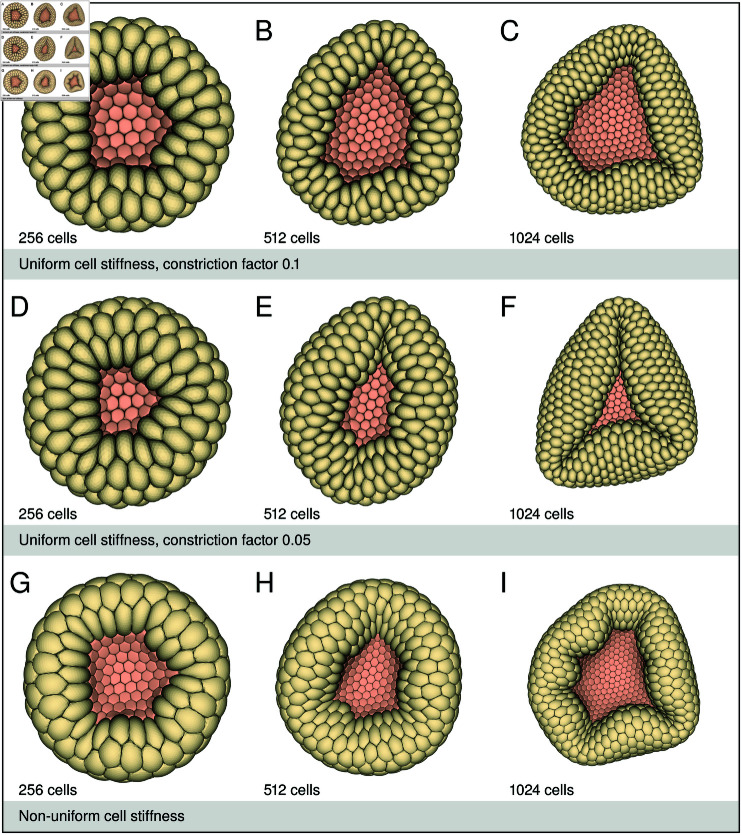
Blastoporal opening. The simulation results show the blastoporal opening of the gastrulas from Fig 5A–5C of the 256–1024 celled gastrulas. Here image A–C show the oral view of the 256, 512 and 1024 celled gastrula, with uniform cell stiffness and constriction factor 0.1. Images D–F show the oral view of the 256, 512 and 1024 celled gastrula, with uniform cell stiffness and constriction factor 0.05. Images G–I show the oral view of the 256, 512 and 1024 celled gastrula, with non uniform cell stiffness. The larger the blastula and the smaller the opening, the more the embryo gets folded around the blastoporal opening.

When the number of endoderm cells in the plate was changed, only the germ layer alignment changed; a high number of endodermal cells could align the germ layers easier, while a low number of endoderm, meant that more ectodermal cells had to move into the blastocoel to get a good fit between the two cell layers. The global gastrula shape after invagination in the simulations, however, was always more or less bowl-shaped with a large opening, independent of the plate shape or cell number, and not spherical, as is seen in biological embryos of for example *Nematostella vectensis*. The global shape in the simulations can be explained by the fact that the blastoporal opening is surrounded by ectoderm cells, that form the future blastopore lip region. The circumferential number of ectoderm cells that form this region, is already larger than the circumferential number of endoderm cells that form the edge of the constricted plate. Therefore, there is an excess of cells in the blastoporal lip region that can resist deformation and prevent the opening from closing. Together with the reduction in the apical endodermal plate area by constriction, these excess cells create folds in the blastoporal lip region (Fig 8C, 8E, 8F, and 8I), preventing closure of the opening. Making the ectoderm in the lip region less stiff, led to more bending and thus better closure of the blastopore opening and helped germ layer alignment. However, the embryo shape remained bowl-like, and viewing the gastrula orally showed an angular appearance (Fig 7B), which is not seen in real embryos. Changing the endodermal plate shape (triangular, star shaped or T-shaped) also helped to reduce the blastoporal opening in the simulations. These shapes created natural folds, that are also seen in *Nematostella vectensis* embryos [10], and reduced the opening. However, in our simulations, these non-spherical plate shapes have large areas inter-spaced with ectoderm that do not constrict apically. This prevented the cells from elongating and establishing contact with the blastocoel wall. So, although alternative plate shapes did reduce the blastoporal opening, it did not change the global embryo shape (which remained bowl-like) or increase the layer alignment.

In the simulations, getting the germ layers to come into complete apposition, while also creating a smaller blastoporal opening and rounder embryo shape, was not possible. Perhaps a different or additional mechanism besides apical constriction and cell-cell adhesion is needed to get a complete spherical gastrula.

## 2D planar versus 3D space simulations

Performing simulations in a 2D plane has some advantages over 3D simulations. On the practical side, planar simulations save time, due to the limited number of cells involved and less cell-cell interactions per cell. When we consider a planar simulation as a cross-section through a 1024 celled blastula, the average simulation time is 10x faster. This increases the number of simulations experiments that can be done in a given amount of time. Besides the practical aspects, planar experiments are easier to interpret, since movements occur in only two directions, instead of three in a full 3D modeled embryo. When comparing the simulation results from the planar cross-sections (S2 Appendix) to the full 3D embryos, we find that, although both can invaginate a cell sheet, the overall embryo shape differs. The planar simulations were able to qualitatively reproduce cellular and global shapes, seen in earlier 2D models [12] and in vivo results, including a closed oral pore, while the 3D simulated embryos remained bowl-like and did not have a closed oral opening. One of the reasons for this difference could be that forces in 2D affect shapes more profoundly and deformations thus are easier to achieve. In 3D simulations, forces are dispersed and not all deformations can be accomplished, since the extra simulated cells create hoop stress that opposes global deformations and the embryo can gain folds as a result (Fig 8C, 8E, 8F and 8I).

This 3D deformable cell based model has shown that simulating mechanical properties in 2D creates qualitatively different shape outcomes compared to 3D simulations. Indicating again, that perhaps we are missing other mechanisms that help the embryo to fully gastrulate and change its shape.

### Outlook.

This research raises several new questions that we would like to explore further. The simulation results showed that the endoderm and ectoderm in the 3D gastrulas did not always come into complete apposition, and thereby did not close the blastocoel completely. These results also showed that the embryo shapes became bowl-like and not spherical with a closed-off blastoporal opening, as seen in *Nematostella vectenis* and *Aurelia aurita*. It is possible that extra mechanisms play a role during invagination in biological organisms. For example, *Nematostella vectensis* and *Aurelia aurita* have a zippering process, where filopodia attach the endodermal cells to the ectoderm, and pull the plate inwards to align and connect the germ layers [11]. Therefore, we are interested to see if the process of zippering can help align and connect the germ layers, and if this mechanism can assist in creating a more spherical embryo after invagination, with a smaller blastoporal opening.

## Conclusion

Our 3D simulations have shown that only a limited number of mechanical cellular properties are required for cell sheet bending: cell-cell adhesion (region), apical constriction factor, cell cortex stiffness and cell volume conservation. These properties are important cellular factors for the shape changes of individual cells. Cell-cell adhesion, which couples the cytoskeleton of cells together, can propagate the pulling forces that the individual cells generate by constricting their cytoskeleton, to neighboring cells. This displaces the cell volume (cytoplasm and nucleus), and depending on the cell cortical stiffness, results in shape changes that bend the cell sheet. The spatial and temporal properties of the embryo determine how the invagination process unfolds and the eventual global shape of the embryo. The shape of the endodermal plate, the number of endodermal versus ectodermal cells in an embryo, the start position of constriction, and the onset of cell constriction, all determine how the endoderm invaginates, how far it can go inwards and if the two germ layers can fully connect.

## Methods

### Cortical elastic elements.

The cortical elements stiffness is length dependent so that stretching is more difficult than compression. This is based on the assumption that on short timescales in a biological cell cortical material is elastic but also limited and thus can not be stretched endlessly (strain stiffening) which gives a nonlinear response [54], while during compression, excess material can be folded into wrinkles.

The *cortical elastic elements* length dependent spring "constant" is described by:

$$F=-k\frac{l}{l_{0}}x$$ (1)

$$x=(l-l_{0}),\;l=x+l_{0}$$ (2)

which can be rewritten to:

$$F=-k\frac{\left(x+l_{0}\right)}{l_{0}}x\;=\;-\frac{k}{l_{0}}x^{2}-kx$$ (3)

Where, *k*: elastic constant (Stiffness), *l*: element length, *l*_0_: rest length, *x*: difference from rest length

### Cortical strain.

In nature, cells can adapt quickly to new cell shapes, free floating cells round up [55], thereby minimizing their surface area, while cells in a blastula can adapt to non-spherical shapes when pushing or pulling forces by neighboring cells are exerted on them through adhesion molecules. Any deviation from the spherical ground state results in an increased surface area created by expansion of the cell cortex. When a cell shape changes from spherical to more elongated, the surface area increases in the cell’s expansion direction and decreases perpendicular to the elongation direction.

When a cell deattaches again it rounds up once more, which means that the biological cell is able to store or use excess material in the cell (cortex) in the form of wrinkles or molecules, or that a quick rearrangement of components can take place [25,26,54,56].

In the model, cells mostly go from spherical to more elongated shapes. These changes need adjustment of the triangulated mesh of the modeled cell, which is facilitated by “cortical strain” [12,13]. This means that the elements are always stretched and try to become shorter, which is opposed by the volume conservation of the cell. For the cortical strain, all edge rest lengths in the modeled cell are factorized at run time, to have a constant tension on the cell to mimic the cells behavior of removing excess cortex material and thereby getting a smooth cell surface. Additional material is generated by stretching the elements.

Due to the conserved cell volume, edges keep tension and they can never reach the equilibrium state where *l* = *l*_*rest*_. Strain on the edges is phrased as follows, each edge “tries” to reach a set rest length. An important effect of this method is, that when the edges of the entire cell are trying to reach a certain “set length” (e.g. 0.25*initial Length) and all edges have the same cell stiffness k, the cell will remain spherical (as seen in a free floating cell) [55].

$$l_{0}=f_{et}l_{rest}$$ (4)

### Constriction.

The cortex can also be actively reshaped by constricting (shortening the rest length of) a designated group of edges, which will automatically lead to expansion of non constricting edges due to volume conservation. To constrict, the edge rest length *l*_0_ is set to a new, shorter, length $l_{c}=f_{c}l_{0}$. Thereby, the edge will try to become the newly designated length, but most probably will never reach it due to the counter acting pulling forces of attached neighboring elements. When the new shape is reached, this shape becomes the new equilibrium shape.

Constriction can be set at any start time on a specific region. The shortening of edges during constriction is done by setting *l*_*rest*_ to its new target length gradually over a certain duration to avoid abrupt changes.

## Volume polyhedron

The simulated cell volume is modeled as an elastic body with the cell cortex as its boundary. When part of a cell is squeezed the volume conservation causes expansion of other parts of the cell by applying forces proportional to the local volume shifts on the cortex so that the cell can adapt its shape.

The volume of each polyhedral cell is found by:

$$V_{polyhedron}=\frac{1}{3}\sum_{face_{i=1}}^{i=n}\vec{x_{i}}\cdot \hat{F_{i}}A_{i}$$ (5)

where *x*_*i*_ is an arbitrary point on the plane of *face*_*i*_, of which the area is represented by the normalized face normal, $\hat{F_{i}}$ times the area, *A*_*i*_.

Volume conservative forces are found by:

$$F_{v}=-k_{v}\frac{V-V_{0}}{V_{0}}$$ (6)

where $F_{v}$ is the directional force per vertex, $k_{v}$: volume stiffness, $V_{0}$: initial volume, *V*: current volume (See S1 Appendix for extended explanation).

## Cell adhesion

**Fig 9:**
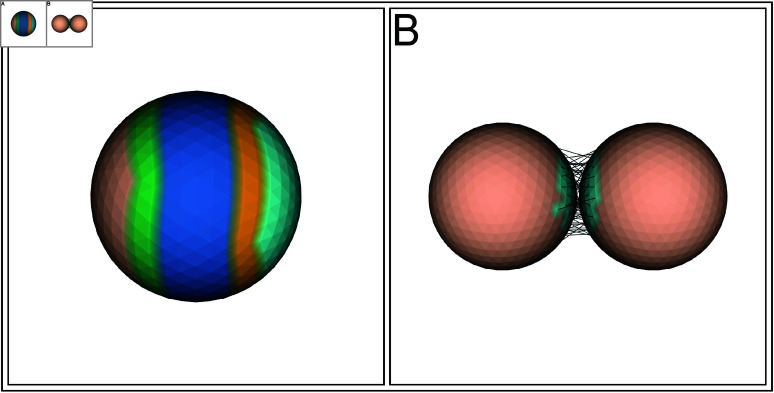
Cell region and adhesion. (**A**) A cell with different regions. The colored bands mark different regions on a cell, which can vary in thickness. (**B**) Cell-cell adhesion. Two cells are adhered together by adhesion molecules (elastic element that connects two vertices) in the selected adhesion region (blue region).

A spherical cell can be divided into regions (Fig 9A). This region can be freely set at the start of the experiment, creating an apical side (outer), lateral side and basal (inner) side with different properties like adhesion or cell stiffness.

The cells of the blastula are adhered together by *cell-cell adhesion*, where an elastic element connects two vertices, each on a different cell (Fig 9B). The adhesive properties can be set per cell and change over time. These include the regions on a cell, the *connect length* and pull strength of the elastic element. The adhesive properties are given to the vertices inside the chosen region and the vertices link when they are within range of the *connect length*. The attached vertices are then pulled together with the chosen force. The adhesive links will pull with a constant force and try to reach a zero length. At zero length the cells will touch. Vertices that are within connect length can connect with a certain chance (adhesion chance and break chance (0-100%), where 0% adhesion chance means no adhesion and 100% adhesion means that every vertex in range will adhere, and are pulled together with the chosen force. The break chance determines of the number of vertices that are attached, how many will disconnect, to reattach to a possible new position.

The elastic element for adhesion is modeled with a constant pulling force, except when arriving close to its target length, *length l* = 0. Then a length dependent linear component (*LinLength*) is included to avoid "bouncing" of the cells due to on/off switching of the pulling force (equation (7). This constant force is based on the assumption that adhesion proteins are connected to the actin cytoskeleton and myosin motors and have a constant pulling force and not behave like springs.

The Constant-Force element is a non continuous function, the behavior is as follows, linear from zero till and including a given length. Thereafter, *F* = *k*_*adh*_.

$$l\leq LinLength,\;F_{adhesion}=-\frac{k_{adh}}{linlength}*l,\;l>LinLength,\;F_{adhesion}=k_{adh}$$ (7)

## Collision

Forming a simulated blastula, by adhering different cells together, requires a way to keep the boundaries of different cells together without being pulled into each other, but still allows for forces to be transferred from one object to another and shape deformations to occur. This is done by collision detection and handling.

**Fig 10:**
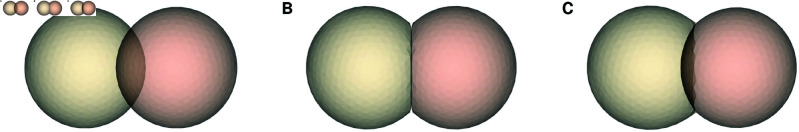
Collision visualization. Collision handling examples of deformable cells: (A) Two overlapping cells, no collision handling. (B) Two equally stiff cells. Collision handled. The stiffness equality causes the cells to deform in the same magnitude. (C) Two cells which have a different stiffness. Collision Handled. The right cell is stiffer than the left cell. The left cell shapes it self around the stiffer right cell. All cell are made transparent to be able to view the effect of the collision handling.

This means that all collisions (boundary violations) must be detected and acted upon accordingly to the laws of physics. Collision is defined as a vertex of one simulated cell crossing the triangular face of the boundary of another body (Fig 10A). When this happens, and is detected, both cells have to locally move their elements out of each others boundaries (Fig 10B and 10C) (see S1 Appendix for extended explanation). Moving out in this case means, that both cells will locally get an opposing force on the violating elements and adjust their shape accordingly to the new situation. Restorative forces are put on both the intruding vertex as well as the three corners of the involved face to ensure local directional force transfer. The magnitude of the restorative force is determined by the perpendicular distance between the vertex and the face (penetration depth). A restorative force will be put on the elements as long as the overlap exists.

The deformable nature of the model makes both the detection and the handling of the collisions a local matter.

## Model dynamics

The system/model dynamics are based on Newton’s second Law of Motion, equation (8), where, *m*, represents the mass, *r*, the displacement and *F*, the total force acting on particle/vertex *i*

$$m_{i}\frac{d^{2}\vec{r_{i}}}{dt^{2}}=\vec{F_{i}}$$ (8)

For each discrete model time step, *t*, the vertex displacement is determined by the total force acting on it, equation (9).

$$\vec{F}=m\vec{a}\;\;or\;\;\vec{a}=\frac{\vec{F}}{m}\;\;where\;\;\vec{a}=\frac{\vec{v}}{t}=\frac{\vec{r}}{t^{2}}\;\;or\;\;\vec{r}=t^{2}\frac{\vec{F}}{m}$$ (9)

The Verlet algorithm (see S1 Appendix) with linear viscous damping, equation (10) is used to determine the new vertex position, *x*_*n* + 1_. Where *c* is the viscous damping factor and *x*_*n*−1_ and *x*_*n*_ the previous and current position.

$${\vec{x}}_{n+1}=(2-c){\vec{x}}_{n}-(1-c){\vec{x}}_{n-1}+\vec{a}\Delta t^{2}$$ (10)

## Optimization

**Fig 11:**
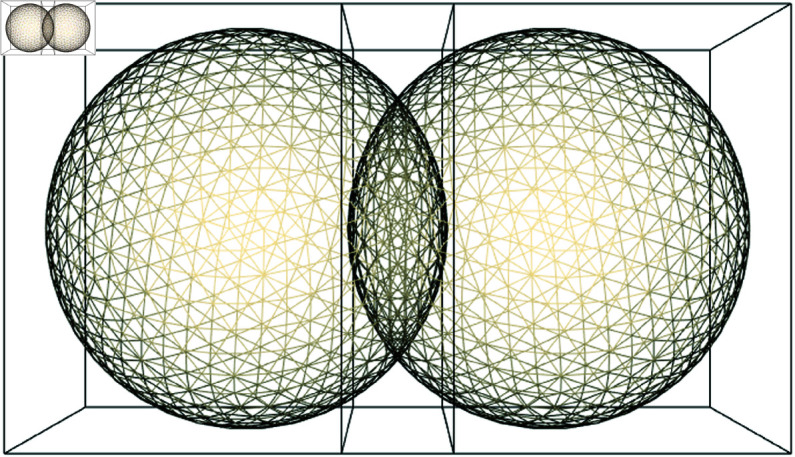
Axis aligned bounding boxes. Two overlapping cells and their axis aligned bounding boxes (AABB). The overlap contains all potentially interacting elements. In this example, the overlap is exaggerated for illustrative purposes. During simulations, the collision detection will have intervened as soon as an overlap is detected and objects will not overlap this much.

Most simulations described here, are done with multiple interacting cells that consist of 642 vertices and 1280 faces. For realistic shape changes, the cells need to deform and transfer forces when they interact. These interactions are adhesion and collision of cells. Determining which vertices and faces of the cells collide and which vertices adhere between objects, are costly operations, considering the amount of potentially interacting elements. Reducing the number of interaction tests, therefore lowers the computational cost. These reductions are done by placing an Axis Aligned Bounding Box (AABB) around the cells (Fig 11). Only boxes that overlap are checked for interactions, and only those element combinations that are in proximity (in the overlapping part) and thus have a chance to interact, are evaluated. This number will be considerably less, than evaluating the total number of elements against each other, To further reduce the number of tests, the overlapping part of the box can be split into smaller sub boxes, which contain has even less elements to test.

**Fig 12:**
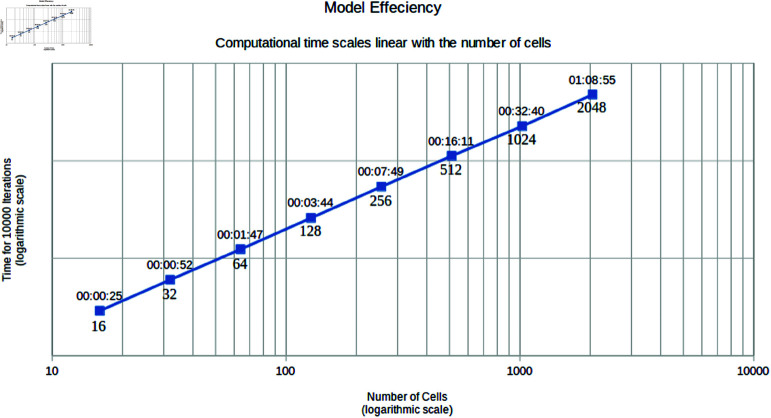
Model efficiency. Model efficiency for blastula simulations with 16-2048 cells. This figure shows the wall clock time (hh:mm:ss) for 1000 iterations when the simulations are in equilibrium state. The time scales linear with the number of cells (axis are logarithmic scale). Doubling the number of cells per blastula results in twice the amount of execution time for the same amount of iterations.

These optimizations, result in a linear relation between the number of cells and the execution time (Fig 12). Although the computational load, especially for the higher cell number simulations, are still high, they are now doable on cpu based systems.

## Planar experiments

Planar experiments are 3D cells placed in a 2D plane. The cells are held in place by two plates, that can be placed at a constant distance (see S2 Appendix).

## Supporting information

S1 Appendix Extended methods. Additional information for the methods.
(PDF)

S2 Appendix Planar simulation results. Planar simulations (3D cells in a 2D plane) that show the effect of individual mechanical parameters on a circular row of cells with 64 cells that simulate a cross section through a blastula. Here the effect of the timing of constriction, constriction factor, adhesion region, cell stiffness and number of endodermal cells on the cell length and the final shape of the gastrula is shown.
(PDF)

S1 Fig Constriction modes in endodermal plate, adhesion region of 20–65%. Time series of endodermal plates with 83 cells. Time interval between constricting cells 500 time units. The shapes that the plates adopted became more extreme.
(PDF)

S2 Fig Constriction modes in endodermal plate, adhesion region of 20–100%. Time series of endodermal plates with 83 cells. Simultaneous constriction, edge cells constrict first, and center cell constricts first. Time interval between constricting cells 100 time units. These results resemble the results with an adhesion region of 20-65% and 100 time units interval between constricting cells.
(PDF)

S3 Fig Cell area. This figure shows the initial round cell, the center ectoderm cell, the center endoderm cell, and a blastoporal lip cell of Fig 5C. The areas increase depending on the shape, which depends on the position in the gastrula.
(PDF)

S1 Graph Apical area graphs. The graphs show the effect of apical constriction in an embryo with a rectangular plate.
(PDF)

S1 Video Movie of Fig 5A. The different blastula sizes (32-1024) with a uniform cell stiffness constrict the endodermal plate.
(MP4)

S2 Video Movie of Fig 7A-7E. Different plate shapes are used for the constricting endodermal plate.
(MP4)

S3 Video Movie of planar simulations of Fig D, S2 Appendix. The constriction factor of the apical area is made smaller for each planar blastula. The largest factor blastula (top left) remains bowl-like. The smallest constriction factor (bottom right) creates a stronger concave shape and the blastula invaginates faster.
(MP4)

S4 Video Movie of planar simulations of Fig G, S2 Appendix. The number of endoderm cells in the planar blastulas is increased, from 8-26. Showing that this influences the alignment of the layers and the filling of the blastocoel space.
(MP4)

S1 Table Parameters used for 3D simulations in Figs 4–5.
(PDF)

S2 Table Parameters used for 3D simulations in Figs 6–7.
(PDF)

S3 Table Parameters used for 3D simulations in Fig 7F–7K.
(PDF)

S4 Table Parameters used for simulations in Fig 3, Figs A,B,D–G, S2 Appendix.
(PDF)

## References

[1] Leptin M. Gastrulation movements: the logic and the nuts and bolts. Dev Cell. 2005;8(3):305–20. doi: 10.1016/j.devcel.2005.02.007 15737927

[2] Technau U. Gastrulation and germ layer formation in the sea anemone Nematostella vectensis and other cnidarians. Mech Dev. 2020;163:103628. doi: 10.1016/j.mod.2020.103628 32603823

[3] Hardin J. The cellular basis of sea urchin gastrulation. Elsevier; 1996, pp. 159–262. 10.1016/s0070-2153(08)60339-7 9138906

[4] Keller R, Davidson LA, Shook DR. How we are shaped: the biomechanics of gastrulation. Differentiation. 2003;71(3):171–205. doi: 10.1046/j.1432-0436.2003.710301.x 12694202

[5] Ettensohn CA. Primary invagination of the vegetal plate during sea urchin gastrulation. Am Zool. 1984;24(3):571–88. doi: 10.1093/icb/24.3.571

[6] Leptin M, Grunewald B. Cell shape changes during gastrulation in Drosophila. Development. 1990;110(1):73–84. doi: 10.1242/dev.110.1.73 2081472

[7] Wen JW, Winklbauer R. Ingression-type cell migration drives vegetal endoderm internalisation in the Xenopus gastrula. Elife. 2017;6:e27190. doi: 10.7554/eLife.27190 28826499 PMC5589415

[8] Hertzler PL. Cleavage and gastrulation in the shrimp Penaeus (Litopenaeus) vannamei (Malacostraca, Decapoda, Dendrobranchiata). Arthropod Struct Dev. 2005;34(4):455–69. doi: 10.1016/j.asd.2005.01.009 19162223

[9] Okubo N, Mezaki T, Nozawa Y, Nakano Y, Lien Y-T, Fukami H, et al. Comparative embryology of eleven species of stony corals (Scleractinia). PLoS One. 2013;8(12):e84115. doi: 10.1371/journal.pone.0084115 24367633 PMC3867500

[10] Kraus Y, Technau U. Gastrulation in the sea anemone Nematostella vectensis occurs by invagination and immigration: an ultrastructural study. Dev Genes Evol. 2006;216(3):119–32. doi: 10.1007/s00427-005-0038-3 16416137

[11] Magie CR, Daly M, Martindale MQ. Gastrulation in the cnidarian Nematostella vectensis occurs via invagination not ingression. Dev Biol. 2007;305(2):483–97. doi: 10.1016/j.ydbio.2007.02.044 17397821

[12] Tamulonis C, Postma M, Marlow HQ, Magie CR, de Jong J, Kaandorp J. A cell-based model of Nematostella vectensis gastrulation including bottle cell formation, invagination and zippering. Dev Biol. 2011;351(1):217–28. doi: 10.1016/j.ydbio.2010.10.017 20977902

[13] Odell GM, Oster G, Alberch P, Burnside B. The mechanical basis of morphogenesis. I. Epithelial folding and invagination. Dev Biol. 1981;85(2):446–62. doi: 10.1016/0012-1606(81)90276-1 7196351

[14] Conte V, Muñoz JJ, Miodownik M. A 3D finite element model of ventral furrow invagination in the Drosophila melanogaster embryo. J Mech Behav Biomed Mater. 2008;1(2):188–98. doi: 10.1016/j.jmbbm.2007.10.002 19627783

[15] Conte V, Ulrich F, Baum B, Muñoz J, Veldhuis J, Brodland W, et al. A Biomechanical analysis of ventral furrow formation in the Drosophila melanogaster embryo. PLoS ONE. 2012;7(4):e34473. doi: 10.1371/journal.pone.0034473 PMC3325263 22511944

[16] Davidson LA, Koehl MA, Keller R, Oster GF. How do sea urchins invaginate? Using biomechanics to distinguish between mechanisms of primary invagination. Development. 1995;121(7):2005–18. doi: 10.1242/dev.121.7.2005 7635048

[17] Polyakov O, He B, Swan M, Shaevitz JW, Kaschube M, Wieschaus E. Passive mechanical forces control cell-shape change during Drosophila ventral furrow formation. Biophys J. 2014;107(4):998–1010. doi: 10.1016/j.bpj.2014.07.013 25140436 PMC4142243

[18] Hočevar Brezavšček A, Rauzi M, Leptin M, Ziherl P. A model of epithelial invagination driven by collective mechanics of identical cells. Biophys J. 2012;103(5):1069–77. doi: 10.1016/j.bpj.2012.07.018 23009857 PMC3433605

[19] Fierling J, John A, Delorme B, Torzynski A, Blanchard GB, Lye CM, et al. Embryo-scale epithelial buckling forms a propagating furrow that initiates gastrulation. Nat Commun. 2022;13(1):3348. doi: 10.1038/s41467-022-30493-3 35688832 PMC9187723

[20] Vasiev B, Balter A, Chaplain M, Glazier JA, Weijer CJ. Modeling gastrulation in the chick embryo: formation of the primitive streak. PLoS One. 2010;5(5):e10571. doi: 10.1371/journal.pone.0010571 20485500 PMC2868022

[21] van der Sande M, Kraus Y, Houliston E, Kaandorp J. A cell-based boundary model of gastrulation by unipolar ingression in the hydrozoan cnidarian Clytia hemisphaerica. Dev Biol. 2020;460(2):176–86. doi: 10.1016/j.ydbio.2019.12.012 31904373

[22] Drasdo D, Forgacs G. Modeling the interplay of generic and genetic mechanisms in cleavage, blastulation, and gastrulation. Dev Dyn. 2000;219(2):182–91. doi: 10.1002/1097-0177(200010)219:2<182::aid-dvdy1040>3.3.co;2-1 11002338

[23] Solnica-Krezel L, Sepich DS. Gastrulation: making and shaping germ layers. Annu Rev Cell Dev Biol. 2012;28:687–717. doi: 10.1146/annurev-cellbio-092910-154043 22804578

[24] Keller R, Shook D. The bending of cell sheets—from folding to rolling. BMC Biol. 2011;9:90. doi: 10.1186/1741-7007-9-90 22206439 PMC3248374

[25] Clark AG, Wartlick O, Salbreux G, Paluch EK. Stresses at the cell surface during animal cell morphogenesis. Curr Biol. 2014;24(10):R484-94. doi: 10.1016/j.cub.2014.03.059 24845681

[26] Lecuit T, Lenne P-F. Cell surface mechanics and the control of cell shape, tissue patterns and morphogenesis. Nat Rev Mol Cell Biol. 2007;8(8):633–44. doi: 10.1038/nrm2222 17643125

[27] Gracia M, Theis S, Proag A, Gay G, Benassayag C, Suzanne M. Mechanical impact of epithelial-mesenchymal transition on epithelial morphogenesis in Drosophila. Nat Commun. 2019;10(1):2951. doi: 10.1038/s41467-019-10720-0 31273212 PMC6609679

[28] Kraus Y, Chevalier S, Houliston E. Cell shape changes during larval body plan development in Clytia hemisphaerica. Dev Biol. 2020;468(1–2):59–79. doi: 10.1016/j.ydbio.2020.09.013 32976840

[29] Heisenberg C-P, Bellaïche Y. Forces in tissue morphogenesis and patterning. Cell. 2013;153(5):948–62. doi: 10.1016/j.cell.2013.05.008 23706734

[30] Janmey PA, Weitz DA. Dealing with mechanics: mechanisms of force transduction in cells. Trends Biochem Sci. 2004;29(7):364–70. doi: 10.1016/j.tibs.2004.05.003 15236744

[31] Botman D, Kaandorp JA. Spatial gene expression quantification: a tool for analysis of in situ hybridizations in sea anemone Nematostella vectensis. BMC Res Notes. 2012;5:555. doi: 10.1186/1756-0500-5-555 23039089 PMC3532226

[32] Kraus Y, Osadchenko B, Kosevich I. Embryonic development of the moon jellyfish Aurelia aurita (Cnidaria, Scyphozoa): another variant on the theme of invagination. PeerJ. 2022;10:e13361. doi: 10.7717/peerj.13361 35607447 PMC9123889

[33] Holcomb MC, Gao G-JJ, Servati M, Schneider D, McNeely PK, Thomas JH, et al. Mechanical feedback and robustness of apical constrictions in Drosophila embryo ventral furrow formation. PLoS Comput Biol. 2021;17(7):e1009173. doi: 10.1371/journal.pcbi.1009173 34228708 PMC8284804

[34] Lye CM, Blanchard GB, Naylor HW, Muresan L, Huisken J, Adams RJ, et al. Mechanical Coupling between Endoderm Invagination and Axis Extension in Drosophila. PLoS Biol. 2015;13(11):e1002292. doi: 10.1371/journal.pbio.1002292 26544693 PMC4636290

[35] Rauzi M, Hočevar Brezavšček A, Ziherl P, Leptin M. Physical models of mesoderm invagination in Drosophila embryo. Biophys J. 2013;105(1):3–10. doi: 10.1016/j.bpj.2013.05.039 23823218 PMC3699736

[36] Marée AFM, Grieneisen VA, Hogeweg P. The cellular potts model and biophysical properties of cells, tissues and morphogenesis. In: Anderson ARA, Chaplain MAJ, Rejniak KA, editors. Single-cell-based models in biology and medicine. Basel: Birkhäuser; 2007, pp. 107–36. doi: 10.1007/978-3-7643-8123-3_5

[37] Okuda S, Inoue Y, Adachi T. Three-dimensional vertex model for simulating multicellular morphogenesis. Biophys Physicobiol. 2015;12:13–20. doi: 10.2142/biophysico.12.0_13 27493850 PMC4736843

[38] Newman TJ. Modeling multicellular systems using subcellular elements. Math Biosci Eng. 2005;2(3):613–24. doi: 10.3934/mbe.2005.2.613 20369943

[39] Alt S, Ganguly P, Salbreux G. Vertex models: from cell mechanics to tissue morphogenesis. Philos Trans R Soc Lond B Biol Sci. 2017;372(1720):20150520. doi: 10.1098/rstb.2015.0520 28348254 PMC5379026

[40] Kumburegama S, Wijesena N, Xu R, Wikramanayake AH. Strabismus-mediated primary archenteron invagination is uncoupled from Wnt/β-catenin-dependent endoderm cell fate specification in Nematostella vectensis (Anthozoa, Cnidaria): Implications for the evolution of gastrulation. Evodevo. 2011;2(1):2. doi: 10.1186/2041-9139-2-2 21255391 PMC3035026

[41] Nathaniel Clarke D, Lowe CJ, James Nelson W. The cadherin-catenin complex is necessary for cell adhesion and embryogenesis in Nematostella vectensis. Dev Biol. 2019;447(2):170–81. doi: 10.1016/j.ydbio.2019.01.007 30629955 PMC6433513

[42] Pukhlyakova EA, Kirillova AO, Kraus YA, Zimmermann B, Technau U. A cadherin switch marks germ layer formation in the diploblastic sea anemone Nematostella vectensis. Development. 2019;146(20):dev174623. doi: 10.1242/dev.174623 31540916

[43] Lee PN, Kumburegama S, Marlow HQ, Martindale MQ, Wikramanayake AH. Asymmetric developmental potential along the animal-vegetal axis in the anthozoan cnidarian, Nematostella vectensis, is mediated by Dishevelled. Dev Biol. 2007;310(1):169–86. doi: 10.1016/j.ydbio.2007.05.040 17716645

[44] Oda H, Tsukita S. Real-time imaging of cell-cell adherens junctions reveals that Drosophila mesoderm invagination begins with two phases of apical constriction of cells. J Cell Sci. 2001;114(Pt 3):493–501. doi: 10.1242/jcs.114.3.493 11171319

[45] Sweeton D, Parks S, Costa M, Wieschaus E. Gastrulation in Drosophila: the formation of the ventral furrow and posterior midgut invaginations. Development. 1991;112(3):775–89. doi: 10.1242/dev.112.3.775 1935689

[46] Leptin M. Mechanics and genetics of cell shape changes during drosophila ventral furrow formation. In: Keller R, Clark WH, Griffin F, editors. Gastrulation. Bodega Marine Laboratory Marine Science Series. Boston, MA: Springer. 1991, pp. 199–212.

[47] Fletcher AG, Osterfield M, Baker RE, Shvartsman SY. Vertex models of epithelial morphogenesis. Biophys J. 2014;106(11):2291–304. doi: 10.1016/j.bpj.2013.11.4498 24896108 PMC4052277

[48] Fletcher AG, Cooper F, Baker RE. Mechanocellular models of epithelial morphogenesis. Philos Trans R Soc Lond B Biol Sci. 2017;372(1720):20150519. doi: 10.1098/rstb.2015.0519 28348253 PMC5379025

[49] Fritzenwanker JH, Genikhovich G, Kraus Y, Technau U. Early development and axis specification in the sea anemone Nematostella vectensis. Dev Biol. 2007;310(2):264–79. doi: 10.1016/j.ydbio.2007.07.029 17716644

[50] Gelbart MA, He B, Martin AC, Thiberge SY, Wieschaus EF, Kaschube M. Volume conservation principle involved in cell lengthening and nucleus movement during tissue morphogenesis. Proc Natl Acad Sci U S A. 2012;109(47):19298–303. doi: 10.1073/pnas.1205258109 23134725 PMC3511084

[51] Hardin J, Keller R. The behaviour and function of bottle cells during gastrulation of Xenopus laevis. Development. 1988;103(1):211–30. doi: 10.1242/dev.103.1.211 3197630

[52] Bhide S, Gombalova D, Mönke G, Stegmaier J, Zinchenko V, Kreshuk A, et al. Mechanical competition alters the cellular interpretation of an endogenous genetic program. J Cell Biol. 2021;220(11):e202104107. doi: 10.1083/jcb.202104107 34449835 PMC8406609

[53] von Dassow M, Strother JA, Davidson LA. Surprisingly simple mechanical behavior of a complex embryonic tissue. PLoS One. 2010;5(12):e15359. doi: 10.1371/journal.pone.0015359 21203396 PMC3011006

[54] Salbreux G, Charras G, Paluch E. Actin cortex mechanics and cellular morphogenesis. Trends Cell Biol. 2012;22(10):536–45. doi: 10.1016/j.tcb.2012.07.001 22871642

[55] Nyga A, Plak K, Kräter M, Urbanska M, Kim K, Guck J, et al. Dynamics of cell rounding during detachment. iScience. 2023;26(5):106696. doi: 10.1016/j.isci.2023.106696 37168576 PMC10165398

[56] Schwarz US, Safran SA. Physics of adherent cells. Rev Mod Phys. 2013;85(3):1327–81. doi: 10.1103/revmodphys.85.1327

