# Supplementary material for: Studying gastrulation by invagination: The bending of a cell sheet by mechanical cell properties using 3D deformable cell based simulations
Source: PLoS Comput Biol. 2025 Jun 25;21(6):e1013151. doi: 10.1371/journal.pcbi.1013151 (PMC12194075; doi:10.1371/journal.pcbi.1013151)
Supplement: S1 Appendix — Additional information for the methods. (PDF) [file pcbi.1013151.s001.pdf]

Supporting information.

S1 Appendix Extended Methods.

Subdivision Icosahedron.

The simulated cells are approximated using geodesic polyhedrons of the class one type. These polyhedrons are created by subdivision of the triangular faces of, in this case, an icosahedron (Fig A). Class one, refers to the method of triangular subdivision; Each triangle of a given polyhedral state is subdivided in to smaller, identical triangles by dividing each edge one or more times into equal lengths. Each division point gets a vertex appointed. The number of edge divisions is referred to as frequencies. A frequency of "1", refers to the non subdivided state of the initial polyhedron, the icosahedron in this case.

When subdividing triangular Face (F), the frequency, f, relates to the number of sub-triangles as:

For the total number of faces of the subdivided polyhedron you get: $F_f = F_i f^2$, where F_i is the initial number of faces on the undivided polyhedron, which is 20 for an icosahedron (See table 1).

The new inserted vertices are connected by edges and are put at the surface of the sphere to make the subdivision complete. An increasing number of subdivisions will result in a better approximation of a perfect sphere, because the detail level increases, but thereby also the number of elements for each cell increases a lot which will result in a higher computational load (Table 1 and Fig B).

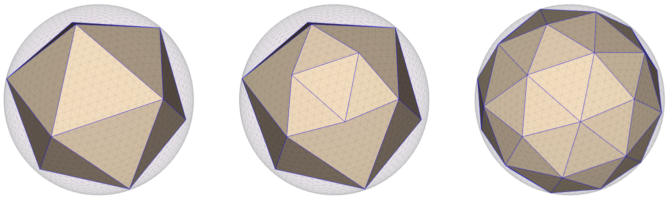

Fig A. Icosahedron subdivision
Example of a frequency "2" subdivision of an icosahedron.

Table 1. Subdivision frequencies
The table gives a series of frequency divisions of an icosahedron. $f = 1$, means no subdivision and thus gives the number of elements found in a icosahedron.

Frequency (f)	f^2	Vertices	Faces	Edges	Elements
1	1	12	20	30	62
2	4	42	80	120	242
3	9	92	180	270	542
4	16	162	320	480	962
5	25	252	500	750	1502
6	36	362	720	1080	2162
7	49	492	980	1470	2942
8	64	642	1280	1920	3842
9	81	812	1620	2430	4862
10	100	1002	2000	3000	6002
11	121	1212	2420	3630	7262
12	144	1442	2880	4320	8642
13	169	1692	3380	5070	10142
14	196	1962	3920	5880	11762
15	225	2252	4500	6750	13502
16	256	2562	5120	7680	15362
17	289	2892	5780	8670	17342
18	324	3242	6480	9720	19442
19	361	3612	7220	10830	21662
20	400	4002	8000	12000	24002

Table notes: frequency 8 is the number of subdivisions used for our simulations.

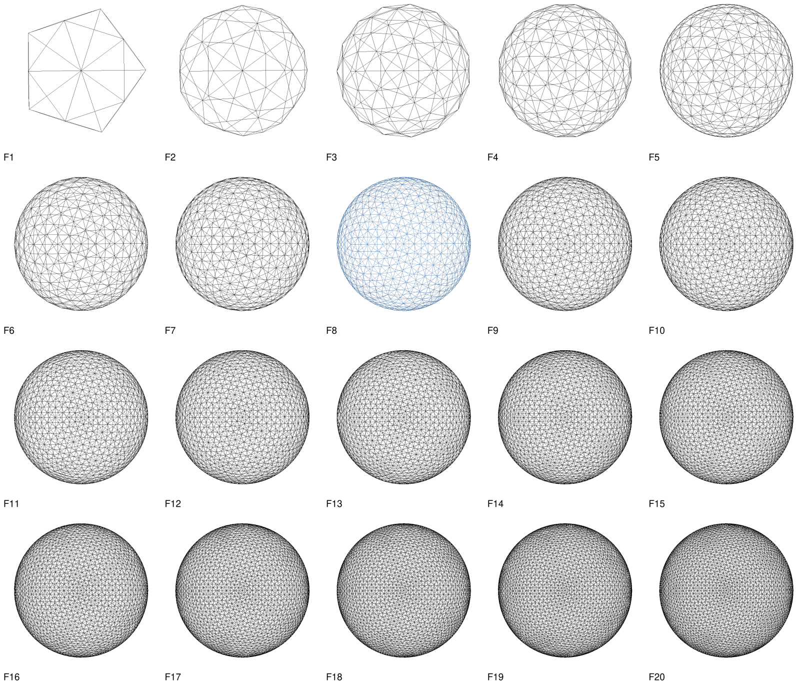

Fig B. Visualized icosahedron subdivisions.
Frequency subdivisions of an icosahedron (top-left Image F1). The frequencies from 1 to 20 are given. In blue the F8 geodesic polyhedron which is used in the presented simulations.

Cell Cortex - Elastic Elements.

Elastic Element.

Other models use Hooke's Law (e.g [1], [2]), but here we use a non-linear element, based on the biological properties of actin networks, which are considered semi-flexible polymers that are cross-linked, causing them to have at short time scales an elastic behavior. Stretching the elements increases the elastic modulus (strain stiffening) and leads to a strong nonlinear behavior [3]. To compare both types of elements we visualized them in Fig C.

Hookes law (equation 2) describes a linear approximation of a spring: Elastic element. This element has a rest length l_0, a length l and their behavior depends on the spring constant k. When the element is either stretched or compressed, the deviation from the rest length is given by x (equation 1). Here, we formulated the elastic elements as a restorative force.

$$x = (l - l_0),\ l = x + l_0 \tag{1}$$

$$F = -kx \tag{2}$$

The elastic energy is given by

$$U(x) = \frac{1}{2}kx^2 \tag{3}$$

For the *cortical elastic elements* used here, we assumed a length dependent spring "constant", which results in that the cortex is easier to compress than to stretch.

$$F = -k\frac{l}{l_0}x \tag{4}$$

rewritten to

$$F = -k\frac{(x + l_0)}{l_0}x \ = \ -\frac{k}{l_0}x^2 - kx \tag{5}$$

To find the elastic energy

$$U(x) = -\int -\frac{k}{l_0}x^2 - kx \ = \ \frac{k}{3l_0}x^3 + \frac{k}{2}x^2 + C \tag{6}$$

for $U(0) = 0$, we find $C = 0$ and thus the elastic energy for the cortical element becomes

$$U(x) = \frac{k}{3l_0}x^3 + \frac{k}{2}x^2 \tag{7}$$

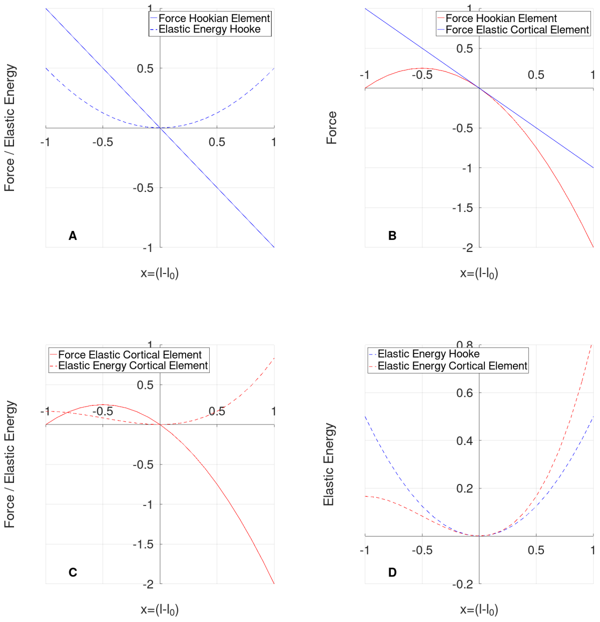

Fig C. Elastic Element Characterization.
Both the force and the elastic energy are plotted against the length difference, x. Compression, $x < 0$, Element at rest, $x = 0$, Stretched, $x > 0$. For all plots, $k = 1$ and $l_0 = 1$. **A)**, Restorative Hookian Element, plotted are the force (solid line) and elastic energy (dashed line) as function of the differential length. **B)**, Comparisson between the force functions of the Restorative Hookian Element (blue line) and the Non-linear Cortical element (red line). **C)**, Non-linear cortical element, plotted are the force (solid line) and elastic energy (dashed line) as function of the differential length. **D)**, Comparison between the elastic energy functions of the Hookian element (blue line) and Non-linear cortical element (red line). The effective compressed length of the non-linear element never becomes smaller than $x > 0.5$.

Volume of a triangulated polyhedron.

To find the volume of a polyhedron, we can make use of the fact that they consist of a finite number of faces, triangles in this case. The triangles are considered as the base of a pyramid which all have their top positioned at the same coordinate, the center of the polyhedron. The sum of the volumes of all the resulting pyramids makes up the total volume of the polyhedron. The volume of a pyramid can be calculated by $\frac{1}{3}$ times height times the area of the base, equation (8). Figure D, shows a polyhedron with a single pyramid highlighted with a triangular face as its base and the top in the center of the polyhedron.

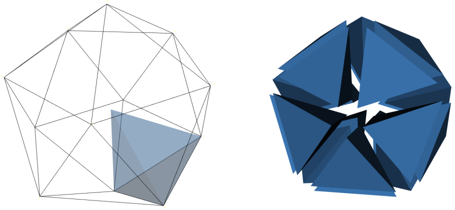

Fig D. Piramids in an icosahedron.
Left image: Icasohedron where for clarity, only one of the 20 pyramids is visualized to determine the volume. Right image: exploded view of all the pyramids making up the icosahedron.

General formula for finding the volume of a given pyramid:

$$V = \frac{1}{3} h A_{base} \tag{8}$$

Following the above principle, a more general expression for volume of a polyhedron can be formulated starting with finding the area of the base of the pyramid (the face triangles). This area is given by the vector norm of the cross-product of two vectors spanning triangle, equation 9 which is depicted in figure E.

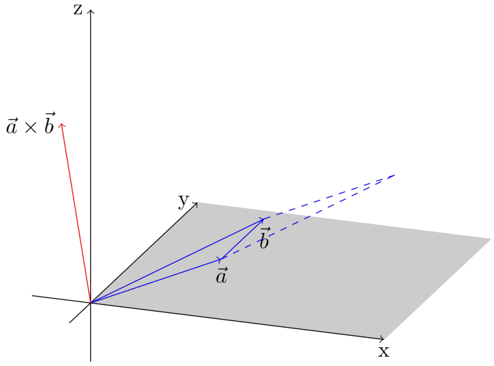

Fig E. Determining the base of the pyramid for the icosahedron volume.
In red, the face normal $\vec{a} \times \vec{b}$ at the origin of the two given vectors, $\vec{a}$ and $\vec{b}$.

Area of a face:

$$A_{face} = \frac{1}{2} \|(\vec{a} \times \vec{b})\| \tag{9}$$

A more general form for the area of a triangle is given by equation 10. This equation is not dependent on the location of the triangle. Equation 9, implicitly assumes that the vectors $\vec{a}$ and $\vec{b}$ originate from the origin.

$$\vec{A}_{face} = \frac{1}{2}((\vec{a} - \vec{c}) \times (\vec{b} - \vec{c})) \tag{10}$$

Area Polyhedron:

$$A_{polyhedron} = \frac{1}{2} \sum_{face_{i=1}}^{i=n} \|((\vec{a_i} - \vec{c_i}) \times (\vec{b_i} - \vec{c_i}))\| \tag{11}$$

Summation over all triangular faces results in the area of the polyhedron.

The height of the pyramid can be found by projection of the top of the pyramid, $\vec{t}$, on to the face normal, a vector perpendicular to the base of the pyramid. The directed area, equation 10, will be used for this. The volume of the pyramid: $\frac{1}{3}$ times the height, times the base area, 8, is given in vectorized form by 12.

$$V = \frac{1}{3}(\vec{t} - \vec{c}) \cdot \frac{1}{2}((\vec{a} - \vec{c}) \times (\vec{b} - \vec{c})) \;\; or \;\; V = \frac{1}{6}(\vec{t} - \vec{c}) \cdot ((\vec{a} - \vec{c}) \times (\vec{b} - \vec{c})) \tag{12}$$

The more general form of equation (12), is known as the scaler triple product which can be interpreted as the volume of a parallelepiped $V = \vec{t} \cdot (\vec{a} \times \vec{b})$. Six triangular based pyramids fit into the parallelepiped, which would be the reverse proof.

The sum over all pyramids gives the volume of the polyhedron [eq 13].

t_i *becomes* t, because it is shared between all pyramids.

$$V_{polyhedron} = \frac{1}{6} \sum_{face_{i=1}}^{i=n} (\vec{t} - \vec{c_i}) \cdot ((\vec{a_i} - \vec{c_i}) \times (\vec{b_i} - \vec{c_i})) \tag{13}$$

A single vertex of the polyhedron could represent the top for each sub volume. In the above examples the top of each pyramid could be imagined to be at the center of mass of the polyhedron. This (shared) position of the top does not have to lie inside the object, it can lie at any arbitrary position, even outside the polyhedron (which can be understood by the signed volumes which are found with the scalar triple product). Basically, the arbitrary position of the top, t, in combination with the fixed position of each base determines the size of the given sub volume of the polyhedron. Thus depending on the position, some pyramids will expand and others will shrink in size compared to another position of t. All the bases of the pyramids form the closed surface area of the polyhedron.

Realizing that the signed sub volumes are proportional to the position of the chosen top and the base of each pyramid, and using the vector pointing at the chosen position t directly, we do not have to subtract c_i to move it to the origin along with $face_i$ to be able to calculate the sub volume. Which would change the term $(\vec{t} - \vec{c_i})$ to $\vec{t}$.

In the same line of thinking, a common top for each sub volume is not necessary, a single point on the same plane as $face_i$ is enough to find the volume of a closed volume. Each sub volume is proportional to the position, length, of the vector resulting from projection of the arbitrary point onto the face normal (cross product) of $face_i$

Choosing vertex c_i for every $face_i$, to serve as reference point, gives equation 14. Vertex c_i was chosen because it is a known point on the surface before moving the base to the origin. Moving all faces to the origin $(\vec{a_i} - \vec{c_i}) \times (\vec{b_i} - \vec{c_i})$ and keeping each c_i on the original position preserves the proportionality and thus the total volume of the object.

$$V_{polyhedron} = \frac{1}{6} \sum_{face_{i=1}}^{i=n} \vec{c_i} \cdot ((\vec{a_i} - \vec{c_i}) \times (\vec{b_i} - \vec{c_i})) \tag{14}$$

Rewriting eq 14, creating a more general form that can be applied to all polyhedrons (independent of the shape of each face), results in eq 15:

$$V_{polyhedron} = \frac{1}{3} \sum_{face_{i=1}}^{i=n} \vec{x_i} \cdot \hat{F}_i A_i \tag{15}$$

Where x_i is an arbitrary point on the plane of $face_i$, of which the area is represented by the normalized face normal, $\hat{F}_i$ times the area, A_i [4].

In the model we state that cells conserve their volume as long as it stays within 98-95% of the initial volume .

Numerical Solver

The displacement of objects due to applied forces are described by Newton's second law of motion [16]. The new position of an object is based on its current position and the applied force. Newton's second law describes the relation between an object with constant mass, m, and its acceleration, $\vec{a}$, due to a given force, $\vec{F}$. The acceleration, velocity per second or distance per second squared gives the variables that are used by the simulator. The relations are given in equations (16) and (17).

Newton's Second law of Motion:

$$m\frac{d^2x}{dt^2} = \vec{F} \tag{16}$$

Finding the displacement:

$$\vec{F} = m\vec{a} \;\; \textbf{or} \;\; \vec{a} = \frac{\vec{F}}{m} \;\; where \;\; \vec{a} = \frac{\vec{v}}{t} = \frac{\vec{x}}{t^2} \;\; \textbf{or} \;\; \vec{x} = t^2\frac{\vec{F}}{m} \tag{17}$$

which states: the directional *force* divided by the the *mass* times a *duration* equals the *displacement.*

Equation 16, monitors the displacement of partials in a continued fashion, e.g. forces, accelerations continuously alter their magnitudes. In the simulator, however, continuous time does not exists. Time progression is done stepwise, discrete steps of finite size (opposed to the infinite small steps made in the continuous formula), thus a discrete approximation for equation 16 is needed. Due to the continuous nature of the physical system which is studied, we have to take care with how to approximate the time progression of the system. The approximation involves, small, but finite progressions in time, dt. What we call small steps are still large compared to continuous functions. The discrete time steps could create problems due to the fact that they could overstep (miss) a change in the system (dt can be seen as the time resolution of the simulated system). Different types of problems can arise from a "to large dt" : Oscillatory behavior, chaotic behavior, or just a slightly different outcome from a simulation with a smaller time resolution. From the last remark we can already conclude that the step size that is best for the simulation is the first one that does not alter the simulation outcome any more. This sounds simple, but it may involve many lengthy simulations to determine this time step and when found, it may not be suitable for a next, but similar, simulation with different parameters.

Implementing the Verlet Algorithm.

The Verlet algorithm was used as numerical solver. The choice was based on the property of being stable on the long term and a low computational load [5].

The Verlet algorithm approximates the future position of an object based on the past and current position (current velocity) and the current acceleration (caused by force) [5]. With this information, the Verlet algorithm is derived with help of Fig F. For the computer simulation we are interested in a fixed finite time step function to find the

next position of the particle based on the force acting on it and the history and current velocity of the particle in mind.

In Fig F the particle positions through time are represented by a dot. Three positions are drawn, past, present and future position: $x_{n-1},\ x_n,\ x_{n+1}$. The time lapse between each new position is equal, the distance traveled not. Because we know the time step size, Δt, and the distance traveled, we can formulate a velocity equation. This equation is build up from past to current, and from current to future time. In equation (18) this part is in the numerator there we are interested in the acceleration.

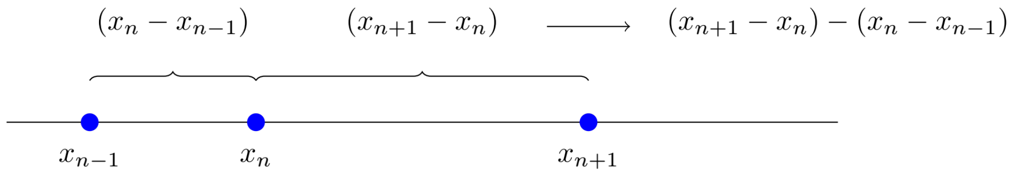

Fig F. Verlet algorithm.
The Verlet algorithm finds the next position for based on the previous and current position. The positions are found in regular, fixed size, time intervals (Δt). Knowing the change in position over time gives the velocity, although it will not be used as such.

$$\vec{a} = \frac{\Delta^2 x}{\Delta t^2} = \frac{\frac{(x_{n+1}-x_n)}{\Delta t} - \frac{(x_n - x_{n-1})}{\Delta t}}{\Delta t} = \frac{-2x_n + x_{n-1} + x_{n+1}}{\Delta t^2} \tag{18}$$

Reordering this equation gives the common form of the Verlet Algorithm:

$$\vec{x}_{n+1} = 2\vec{x}_n - \vec{x}_{n-1} + \vec{a}\Delta t^2 \tag{19}$$

Equation 17 gives how the acceleration term, $\vec{a}$, follows from the forces in the system.

Here we start the simulations in equilibrium state, and thus the previous state is known and equals the current location x_{n-1}.

Viscous Damping.

Adding a linear viscous damping term, which is proportional to the current velocity : cv completes the numerical solver used in the simulations.

Linear Viscous Damping Term:

$$-cv = -c\frac{(x_n - x_{n-1})}{\Delta t} \quad \textit{where} \quad 0 \leq c \leq 1 \tag{20}$$

Combining equation (18) and (20) gives a similar form as equation (19).

$$\vec{x}_{n+1} = 2\vec{x}_n - \vec{x}_{n-1} + \vec{a}\Delta t^2 - c(\vec{x}_n - \vec{x}_{n-1}) \tag{21}$$

The numerical solver with linear viscous damping can be rewritten into:

$$\vec{x}_{n+1} = (2-c)\vec{x}_n - (1-c)\vec{x}_{n-1} + \vec{a}\Delta t^2 \tag{22}$$

Maximum damping occurs with $c = 1$, in this situation the current velocity is canceled out by the viscous damping and thus this situation could be referred to as an inertialess system.

References

1. Tamulonis C, Postma M, Marlow HQ, Magie CR, de Jong J, Kaandorp J. A cell-based model of Nematostella vectensis gastrulation including bottle cell formation, invagination and zippering. Dev Biol. 2010;351(1):217–228. doi:10.1016/j.ydbio.2010.10.017.

2. Odell GM, Oster G, Alberch P, Burnside B. The mechanical basis of morphogenesis. I. Epithelial folding and invagination. Dev Biol. 1981;85(2):446–462. doi:10.1016/0012-1606(81)90276-1.

3. Salbreux G, Charras G, Paluch E. Actin cortex mechanics and cellular morphogenesis. Trends Cell Biol. 2012;22(10):536–545. doi:10.1016/j.tcb.2012.07.001.

4. Goldman RN. In: Arvo J, editor. Area of planar polygons and volume of polyhedra, IV.1. San Diego: Morgan Kaufmann; 1991. p. 170–171.

5. Frenkel D, B S. 4. In: Molecular Dynamics Simulations. 2nd ed. USA: Academic Press, Inc; 2002. p. 63–107.
