# Supplementary material for: Studying gastrulation by invagination: The bending of a cell sheet by mechanical cell properties using 3D deformable cell based simulations
Source: PLoS Comput Biol. 2025 Jun 25;21(6):e1013151. doi: 10.1371/journal.pcbi.1013151 (PMC12194075; doi:10.1371/journal.pcbi.1013151)
Supplement: S2 Appendix — Planar simulations (3D cells in a 2D plane) that show the effect of individual mechanical parameters on a circular row of cells with 64 cells that simulate a cross section through a blastula. Here the effect of the timing of constriction, constriction factor, adhesion region, cell stiffness and number of endodermal cells on the cell length and the final shape of the gastrula is shown. (PDF) [file pcbi.1013151.s002.pdf]

Supporting information.

S2 Appendix Planar invagination simulations

2D models have shown that mechanical cellular properties determine the local cell shape and the global embryo shape during the invagination process [1, 2]. These models found, that apical constriction and cell-cell adhesion are essential properties for invagination to take place, We used 3D cells in a 2D plane (planar simulations) to test the effect individual cellular properties have on the cell shape and the embryo shape, and used these results to validate the model and to compare it qualitatively to 2D models. The blastula was modeled, so as to resemble a cross section through a spherical 3D embryo consisting of approximately 1024 cells. This simulated blastula consisted of 64 3D deformable cells placed in a circle and adhered together. The cells were held between two virtual plates that kept the cells in the simulated plane. Due to the limited number of cells, these simulations were computationally less heavy.

Of the 64 simulated cells between 8 and 26 cells were appointed as endodermal cells by choosing the parameters as such that they could act as endoderm: the triangulated mesh could constrict apically (edges try to become shorter) and adhesion molecules at the lateral sides were disconnected to allow for more lateral movement.

The cell stiffness for most experiments consisted of a high apical stiffness (k=1.8) and a low basal stiffness (k=0.1), unless otherwise mentioned. In the simulation results, the endodermal cells are colored salmon and the ectodermal cells beige.

The parameters of the experiments are given in S4 Table.

Constriction time interval

Endodermal plate shapes can initially be flat, bowl shape or even concave as seen during the constriction process (Fig 2J and K main text). The hypothesis is that this is due to a difference in timing between the constricting cells. To test this, we varied the timing between constricting cells. From no time between constriction (simultaneous constriction), to 1000 time units between constricting cells. Simultaneous constriction, where all endodermal cells are adhered together and constrict apically with no time interval between constricting cells caused first an inward movement of the edge of the endodermal plate attached to the ectodermal cells and an outward movement of the top of the endodermal plate (convex shape). The plate then flattened out (T=30), before it moved inwards (Fig A shows a time series). Time step T=50 resembles the flattened plate seen in stony coral (Fig 2J main text).

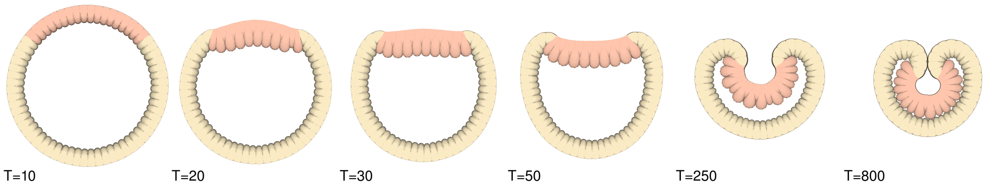

Fig A. Simultaneous apical constriction of endodermal cells.
64 Cells form a planar blastula with 18 endoderm cells. Simulation time points are indicated with T. Constricting the endodermal cells (salmon) simultaneously caused the edge of the endodermal plate to move inwards first and buckle the center of the plate upwards (convex shape), then flatten out, before moving inwards.

Simultaneous constriction in virtual cells resembles the flattened plate seen in Stony corals (Fig 2J main text). However, in *Nematostella vectensis* [3] and *Aurelia aurita* [4] constriction appears to start approximately at the center of the plate, creating a concave indent (Fig 2K main text), after which the constricted area increases with time.

Simulating this ring-like constriction, seen in *Nematostella vectensis* embryos, in a planar simulation, means that the center cells starts the constriction process followed by the neighboring cells, which constrict one by one. A time interval between these constricting cells creates a constriction wave that moves to the edge of the constricting region. In 2D this creates a symmetrical pattern along the oral-aboral axis. We found that increasing the time interval from 500 to 1000 time units between constricting cells made it possible for the cells to move further into the blastocoel and the endodermal plate to become more concave (see Fig B) during the invagination process. Constricting the first cells, caused them to change shape and to start moving with a certain velocity. If the next constricting cells followed soon after the first, this action opposed the force created by the first constricting cells. Causing the inward displacement of these cells to halt, and the endodermal plate to level out earlier, leaving the endodermal plate and the shape of the embryo to become temporarily flatter. To emphasize the effect, the number of endodermal cells was increased from 18 to 26.

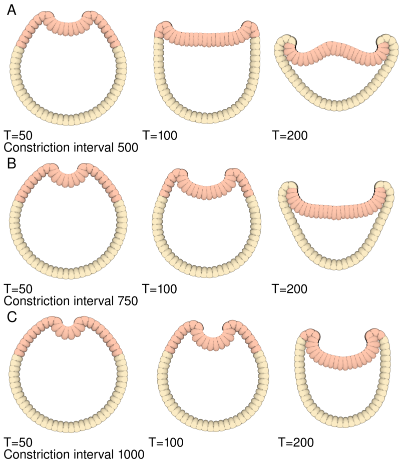

Fig B. Time interval between constricting cells.
64 Cells form a 2D blastula with 26 endoderm cells. Constriction takes place cell by cell, representing ring constriction, with a certain time interval between them before the next cells constrict.
Time interval before the next cell constricts: row A). 500 time units, row B). 750 time units, row C). 1000 time units, at simulation time points T=50, T=100, and T=200.
To emphasize the time constriction effect, the cell stiffness was changed. The apical region (0-30%) stiffness k=1, the lateral region (30-70%) stiffness k=0.5%, basal region (70-100%) stiffness k=0.1.
Increasing the time between constricting rings from 500 to 750 and 1000 time units, made the endodermal plate more concave and invagination faster. It also influenced the global shape of the embryo, changing it from bowl-like (Image A, T=200) to more elongated (Image C, T=200).

Measuring embryo dimension in 2.5D simulation results.

The embryo dimensions in 2D simulation results were measured using a contour box (Fig C), to determine the width and height of the embryo. The archenteron and blastocoel dimensions are determined at the widest area. The endodermal cell height is only determined at the oral-aboral axis. The ectodermal cell heights are determined laterally and on the oral-aboral axis. These measurements were used in the graphs below to determine differences between the results of the different experiment parameters.

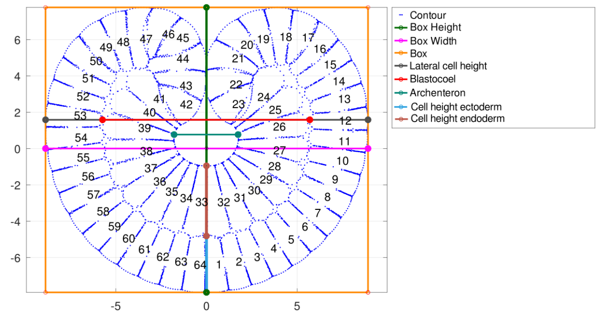

Fig C. Measuring method to determine width and heights of embryo.
Around the 2D embryo is placed a box to determine the width and height of the embryo. The archenteron and blastocoel dimensions are determined at the widest area. The endodermal cell height is only determined at the oral-aboral axis. The ectodermal cell heights are determined laterally and on the oral-aboral axis

Constriction factor

During constriction the apical area of an endodermal cell reduces significantly, which displaces the cell volume and moves the nucleus basally. Resistance from neighboring cells can then cause the cell to elongate into a bottle-shape and bend the endodermal plate [3].

In our model the apical edge constriction factor determines how small the constricting apical edges try to become. In reality, the cell will probably never reach the new edge length, due to pulling forces from neighboring cells that oppose the constriction force. The apical edge constriction factor of single cells, will eventually determine the apical area and the bending of the endodermal plate (Fig D image A-F and S3 Video). A blastula with a small apical edge constriction factor (Fig D image F) meant that the apical edges became smaller, than with a larger edge constriction factor (Fig D image A). This caused the endodermal plate to buckle inwards faster and with a tighter curvature that increased the concave shape of the plate (Fig D image F). It also increased the endodermal cell height and decreased the archenteron opening, as is seen in the graphs of the blastula and cell length (Fig D) (See Fig C for measurement method).

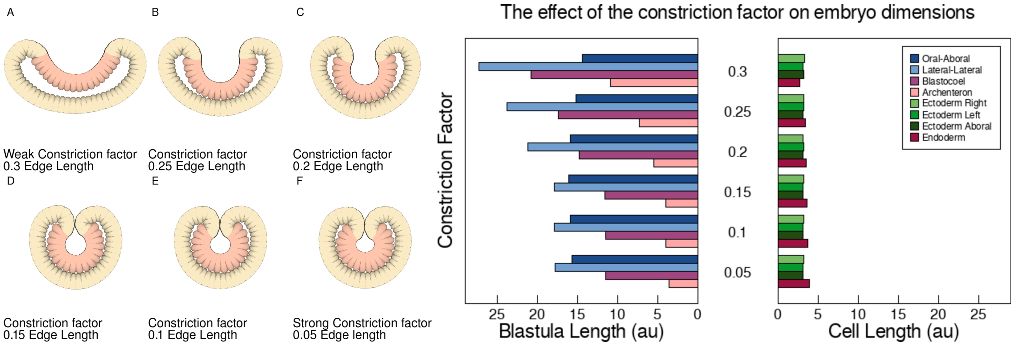

Fig D. Constriction factor.
64 Cells form a 2D blastula with 18 endoderm cells. Constriction factor in image goes from large to small, where: image A). 0.3, B). 0.25, C). 0.2, D). 0.15, E). 0.1, F). 0.05. A large edge constriction factor (figure A) caused a more flattened shape of the endodermal plate and global embryo shape, whereas a smaller edge constriction factor (figure F) led to an almost spherical embryo.
More constriction (smaller edge) caused a smaller apical surface which translated in a stronger concave global curve of the endodermal plate, an increase in endodermal cell length, and decrease in archenteron opening size, as seen in the graph.

Adhesion region

For gastrulation to begin, the epithelialized blastula needs to partially de-epithelialize (loose adhesion), only retaining the adhesion junctions located apically [1]. Constriction then displaces the internal volume, causing bottle cells to form and the endodermal plate to invaginate [1].

Reducing the adhesion region between endodermal cells, from 90% to 60% (Fig E row A), influenced the lateral movement of these cells. The small apical adhesion band allowed cells to laterally slide over each other, and shape changes to emerge due to forces from neighboring cells. Some cells became bottle shaped, while others were suppressed by these elongated cells into squat cells. The images (Fig E row A) show that with 60% adhesion at time point T=250, many squat cells were present, but finally most cells elongated again at time point T=500, when invagination was complete (cells are made transparent for clarity).

Fully attached cells (0-90% adhesion) (Fig E row B), all elongated after constriction and only showed squat like cells in the unconstricted cells. The adhesion prevented lateral movement. The global embryo properties however, did not differ much between the adhesion region experiments, as is seen in the graphs.

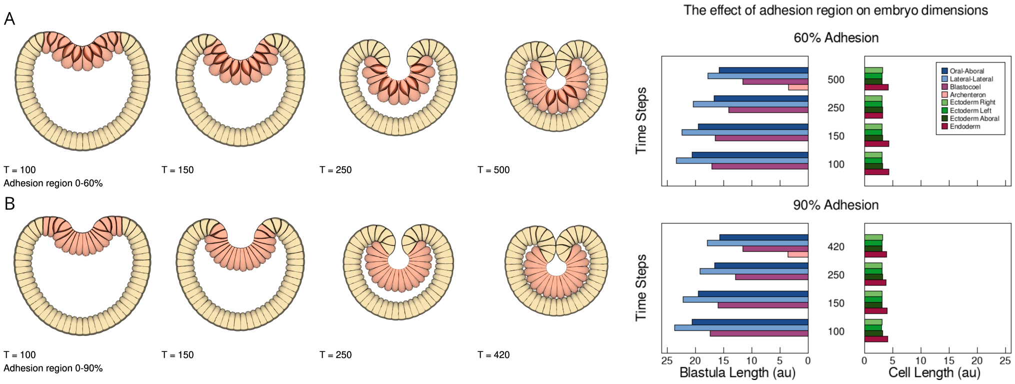

Fig E. Adhesion region

Two time series of blastulas with 64 cells, that form a 2D blastula with 18 endoderm cells. Top row A): Adhesion region 60%. Bottom row B): Adhesion region 90%.

For clarity, the gastrulas have been made transparent. An adhesion region of 60% of the spherical cell (top row) gave more lateral movement to cells and cell shape differentiation was seen; bottle and squat cell shapes emerged during invagination. These shapes changed again during invagination; squat cells changed into bottle cell shapes. Where as, when the endodermal cells were more adhered to each other (90% adhesion, bottom row), the cells became uniformly longer and squat cells were only observed in the bend regions, alongside the constricting cells. After all the endodermal cells were constricted no squat cell shapes remained. The graphs show that at time point T=250 with 60% adhesion the endodermal cells (maroon bar) decreased in length, before finally increasing again at T=500. This was not the case for the gastrula with 90% adhesion.

Cell stiffness

During constriction, the apical area of cells is assumed to be stiffer than the lateral and basal area of a cell in order for the embryo to be in a mechanical equilibrium [1].

Adding a large difference in apical and basal cell stiffness (Fig F image A), created longer and narrower cells, causing the diameter of the total embryo and the archenteron to become smaller and the global shape rounder. 2D blastulas where the difference between the apical and basal side of the cells was smallest, had shorter cells and a large gastrula and archenteron opening (Fig F image H).

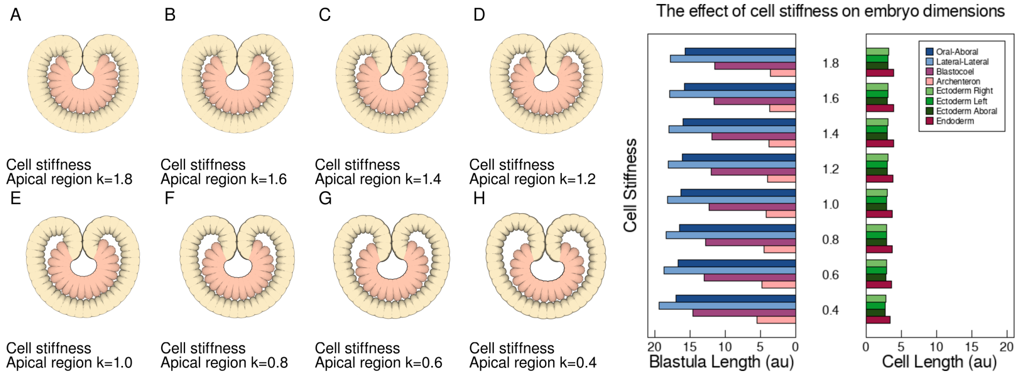

Fig F. Endoderm cell stiffness difference apically and basally.
This figure shows the difference in cell stiffness between the apical and basal side of the cell (for both the endodermal and ectodermal cells). 64 Cells form a 2D blastula with 18 endoderm cells. The apical top region 0-40% of the spherical cell, had the greatest cell stiffness, the basal region 40-100% had the lowest cell stiffness. Apical-basal cell stiffness difference image A). 1.8-0.1, B). 1.6-0.1, C). 1.4-0.1, D). 1.2-0.1, E). 1.0-0.1, F). 0.8-0.1, G). 0.6-0.1, H). 0.4-0.1. The apical constriction factor for the endodermal cells is 0.05 for all blastulas. A bigger difference between the apical and basal stiffness caused the cells to elongate more, which assisted in the infolding of the endodermal plate into the blastocoel and led to a more spherical embryo shape with a smaller archenteron (image A).
Invagination occurred in all eight blastulas. In gastrulas where the apical-basal difference was smallest (image H), the endodermal plate only partially aligned with the ectoderm, and the archenteron opening became larger. There was no alignment closest to the blastoporal opening.

Number of endoderm cells

In *Nematostella vectensis* the number of endodermal cells is approximately a fourth or a fifth of the blastula [1,3]. Images of Stony corals [5] and *Aurelia aurita* [4] invagination, show similar ratios of endodermal versus ectodermal cells. For a 64 celled 2D blastula this would result in 13-16 endoderm cells. Fig G shows that, the number of endodermal cells determines if an embryo after invagination can align its germ layers or not (movie S4 Video). Increasing the number of endodermal cells (from 8 to 26), influenced the invagination process. Too few endodermal cells (Fig G image A), prevented the endodermal plate to align with the ectoderm and the embryo shape became less spherical. Too many endodermal cells prevented the blastopore opening from closing, since an increase in endoderm automatically meant a decrease in ectoderm cells, reducing the blastocoel space. The blastulas with 20, 22 and 24 endodermal cells resemble each other closely. However, the movie shows that the speed with which they invaginated was fastest for 20 endodermal cells.

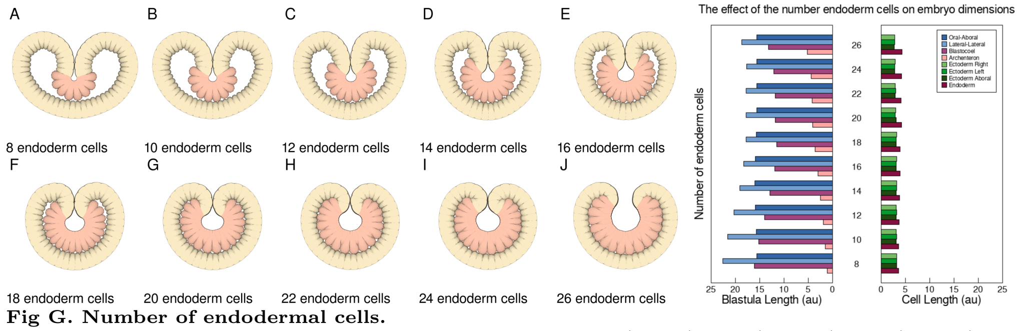

Fig G. Number of endodermal cells.
64 Cells form a 2D blastula. Number of endodermal cells: Image A). 8, B). 10, C). 12, D). 14, E). 16, F). 18, G). 20, H). 22, I). 24, J). 26. The number of endodermal cells influences the invagination process. The more endodermal cells that were assigned to the endodermal plate, the greater the area became that contained connected cells. Also the archenteron opening became larger with more cells and the total embryo more spherical (see graph). For blastulas with 20 endodermal cells or less, alignment always started opposite the blastoporal opening, but for 22 endodermal cells or more, alignment of the endodermal cells with the ectodermal cells started at the lateral sides before the entire plate moved downwards. More than 24 endodermal cells prevented the archenteron opening from closing and widened the embryo again.

References

1. Tamulonis C, Postma M, Marlow HQ, Magie CR, de Jong J, Kaandorp J. A cell-based model of Nematostella vectensis gastrulation including bottle cell formation, invagination and zippering. Dev Biol. 2010;351(1):217–228. doi:10.1016/j.ydbio.2010.10.017.

2. Odell GM, Oster G, Alberch P, Burnside B. The mechanical basis of morphogenesis. I. Epithelial folding and invagination. Dev Biol. 1981;85(2):446–462. doi:10.1016/0012-1606(81)90276-1.

3. Magie CR, Daly M, Martindale MQ. Gastrulation in the cnidarian Nematostella vectensis occurs via invagination not ingression. Dev Biol. 2007;305(2):483–497. doi:10.1016/j.ydbio.2007.02.044.

4. Kraus Y, Osadchenko B, Kosevich I. Embryonic development of the moon jellyfish Aurelia aurita (Cnidaria, Scyphozoa): another variant on the theme of invagination. PeerJ. 2022;10:e13361. doi:10.7717/peerj.13361.

5. Okubo N, Mezaki T, Nozawa Y, Nakano Y, Lien YT, Fukami H, et al. Comparative embryology of eleven species of stony corals (Scleractinia). PLoS One. 2013;8(12):e84115. doi:10.1371/journal.pone.0084115.
