# Supplementary material for: Studying gastrulation by invagination: The bending of a cell sheet by mechanical cell properties using 3D deformable cell based simulations
Source: PLoS Comput Biol. 2025 Jun 25;21(6):e1013151. doi: 10.1371/journal.pcbi.1013151 (PMC12194075; doi:10.1371/journal.pcbi.1013151)
Supplement: S1 Fig — Time series of endodermal plates with 83 cells. Time interval between constricting cells 500 time units. The shapes that the plates adopted became more extreme. (PDF) [file pcbi.1013151.s003.pdf]

Supporting information.

S1 Fig. Constriction modes in endodermal plate, additional results.

In Fig 1 detached endodermal plates were constricted with an adhesion region of 20-65% but now the time interval between constricting cells was 500 time units instead of 100 time units (Fig 4A-C in main text). The shapes that the plates adopted became more extreme; with the edge cells constricting first, the edge cells curled more tightly around the convex center of the plate. The center cell constricting first method (second row) showed that the invaginating shape becomes more concave.

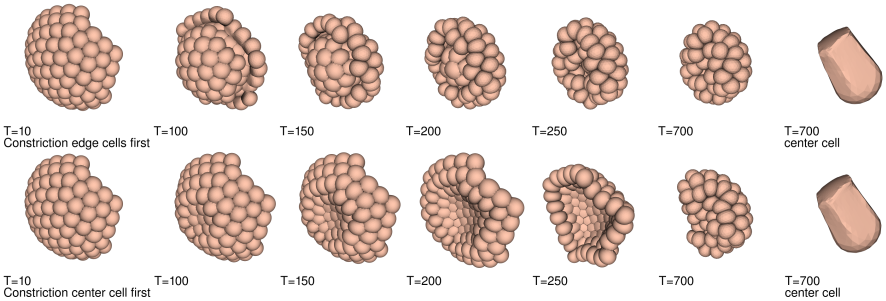

Fig 1. Constriction methods in plates with 20-65% adhesion.
Time series of endodermal plates with 83 cells. Row 1: Edge cells constrict first with time interval between constricting cells 500 units, row 2: center cell constricts first with time interval between constricting cells 500 units. Cell stiffness k=0.5, adhesion region 20-65% and constriction factor 0.1, time steps 10, 100, 150, 200, 250, 700
