# Supplementary material for: Studying gastrulation by invagination: The bending of a cell sheet by mechanical cell properties using 3D deformable cell based simulations
Source: PLoS Comput Biol. 2025 Jun 25;21(6):e1013151. doi: 10.1371/journal.pcbi.1013151 (PMC12194075; doi:10.1371/journal.pcbi.1013151)
Supplement: S2 Fig — Time series of endodermal plates with 83 cells. Simultaneous constriction, edge cells constrict first, and center cell constricts first. Time interval between constricting cells 100 time units. These results resemble the results with an adhesion region of 20-65% and 100 time units interval between constricting cells. (PDF) [file pcbi.1013151.s004.pdf]

Supporting information.

S2 Fig. Constriction modes in endodermal plate, additional results.

Fig 1 shows endodermal plates with an adhesion region of 20-100% and time interval of 100 units between constricting cells. These result resemble the results in Fig 4A-C in main text. Showing that the effect of the adhesion region does not notably influence the bending of the plate.

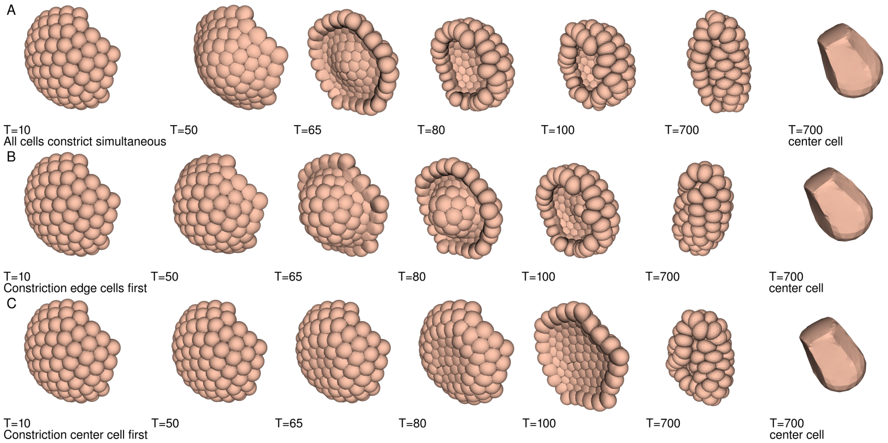

Fig 1. Constriction methods in plates with 20-100% adhesion.
Time series of endodermal plates with 83 cells. Row 1:simultaneous constriction, row 2: Edge cells constrict first with time interval between constricting cells 100 units, row 3: center cell constricts first with time interval between constricting cells 100 units. Cell stiffness k=0.5, adhesion region 20-100% and constriction factor 0.1, time steps 10, 50, 65, 80, 100, 700
