# Supplementary material for: Studying gastrulation by invagination: The bending of a cell sheet by mechanical cell properties using 3D deformable cell based simulations
Source: PLoS Comput Biol. 2025 Jun 25;21(6):e1013151. doi: 10.1371/journal.pcbi.1013151 (PMC12194075; doi:10.1371/journal.pcbi.1013151)
Supplement: S3 Fig — This figure shows the initial round cell, the center ectoderm cell, the center endoderm cell, and a blastoporal lip cell of Fig 5C. The areas increase depending on the shape, which depends on the position in the gastrula. (PDF) [file pcbi.1013151.s005.pdf]

Supporting information.

S3 Fig. Cell area.

Cells that adhere together loose their spherical shape and can increase their cell surface area by 50% [1–3]. Animal cells that do not adhere will tend to round up, due to the surface tension created by the plasma membrane and actin cortex. The adhesion and constriction of cells positioned in the endodermal plate area, changes the shape of these cells from spherical to wedge and eventually elongated. However, the local cell shape changes not only affect the endodermal plate, which bends and invaginates, but also changes the shape of the ectodermal cells in and around the blastoporal opening. These cortical shape changes, must be accommodated by the lipid plasma membrane to allow for an increased surface area. This is a very dynamic process, where excess membrane can be stored as wrinkles and folds, or added as new material if necessary [1, 4, 5]. Below we compare the cell shapes from cells found at different locations in the same gastrula to a spherical unattached cell (Fig 5C in main text). These cells all have a different surface area. A round cell has an area of 1, while the area of the ectodermal, endodermal and blastoporal cell increase respectively.

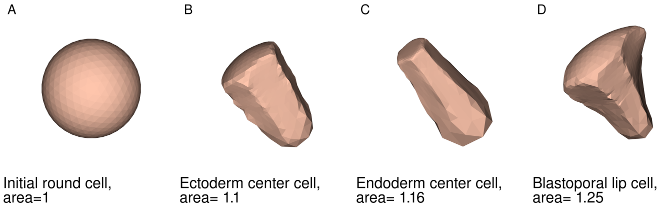

Fig 1. Cell areas of Fig 5C
This image compares cell areas of cells at different positions in the 256 celled gastrula of Fig5C to a spherical cell: A). Initial round cell, area=1. B). Center ectodermal cell, area=1.1. C). Center endodermal cell, area=1.16. D) Blastoporal lip cell, area=1.25 of Fig 5C, 256 celled blastula.
The cell areas of the cells at different positions in the gastrula are compared to the round cell (image A). The center aboral ectodermal cell (image B) is only adhered to neighboring cells, while the endodermal cell also constricts apically (image C). The area of the blastoporal lip cell (image D) increases most since this cell is on the edge of the active constricting endodermal cells, and the passive deforming ectodermal cells.

References

1. Schwarz SSA Ulrich S. Physics of adherent cells. Rev Mod Phys. 2013;85:1327–1381. doi:10.1103/RevModPhys.85.1327.

2. Paluch E, Heisenberg CP. Biology and physics of cell shape changes in development. Curr Biol. 2009;19(17):R790–9. doi:10.1016/j.cub.2009.07.029.

3. Nyga A, Plak K, Kräter M, Urbanska M, Kim K, Guck J, et al. Dynamics of cell rounding during detachment. iScience. 2023;26(5):106696. doi:10.1016/j.isci.2023.106696.

4. Lecuit T, Lenne PF. Cell surface mechanics and the control of cell shape, tissue patterns and morphogenesis. Nature Reviews Molecular Cell Biology. 2007;8(8):633–644. doi:10.1038/nrm2222.

5. Clark AG, Wartlick O, Salbreux G, Paluch EK. Stresses at the cell surface during animal cell morphogenesis. Curr Biol. 2014;24(10):R484–94. doi:10.1016/j.cub.2014.03.059.
