# Supplementary material for: Studying gastrulation by invagination: The bending of a cell sheet by mechanical cell properties using 3D deformable cell based simulations
Source: PLoS Comput Biol. 2025 Jun 25;21(6):e1013151. doi: 10.1371/journal.pcbi.1013151 (PMC12194075; doi:10.1371/journal.pcbi.1013151)
Supplement: S1 Graph — The graphs show the effect of apical constriction in an embryo with a rectangular plate. (PDF) [file pcbi.1013151.s006.pdf]

Supporting information.

S1 Graphs Apical area

Fig 1 shows the rows in the blastula of Fig 6A in main text. The center row is colored red. The rows above and below: row 1, purple, row 2, blue, row 3, light green, row 4, aquamarine blue. In each row, 4 cells are indicated by their id number. These cells are measured by taking 2 vertices on a horizontal line and 2 vertices vertically and measuring the distance between them every time step. These lengths are plotted in the graphs below (Fig 2). The left graphs give the area length vertically measured, in the top figure the rows above the center rows are visualized and in the bottom figure, the rows below the center row are visualized. The right graphs give the horizontal measurements. Top graph: again rows above the center row, bottom graph: rows below the center row. The lines are colored according to the row colors used in Fig 1. The left figures (vertical length) show that the center row starts constriction at time step 50, and reaches its smallest length at time step 100. It then slowly increases again in a wave or pulsed pattern. This pulsed increase corresponds with the start of each next constricting row. The constriction of each following row inhibits the constriction of the row before it and pulls on the apical area, slightly stretching it again. It also pulls on the apical area of the rows that have not yet constricted, increasing their area, and creating a peak before it starts to constrict. This causes the center row and first constricting row to have the largest vertical length and the last row to be able to constrict the most, since now there are now more rows that constrict, only the ectoderm that is passively stretched. This pattern is more or less similar between the graphs of the rows above and the rows below. Horizontally, the center row cells have the largest length, that slowly decreases. The following rows become smaller with each next row, repeating the final length pattern seen in the vertical length graph at time step 700. Here, with each constricting row the horizontal length decreases since now the rows before have pulled the cells compacter.

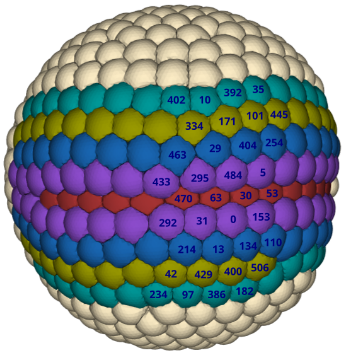

Fig 1. Measured cells.
This image shows the rows in the blastula of Fig 6A in main text. The center row is colored red, the rows above and below, row 1: purple, row 2: blue, row 3: light green, row 4: aquamarine blue. In each row, 4 cells are indicated by their id number.

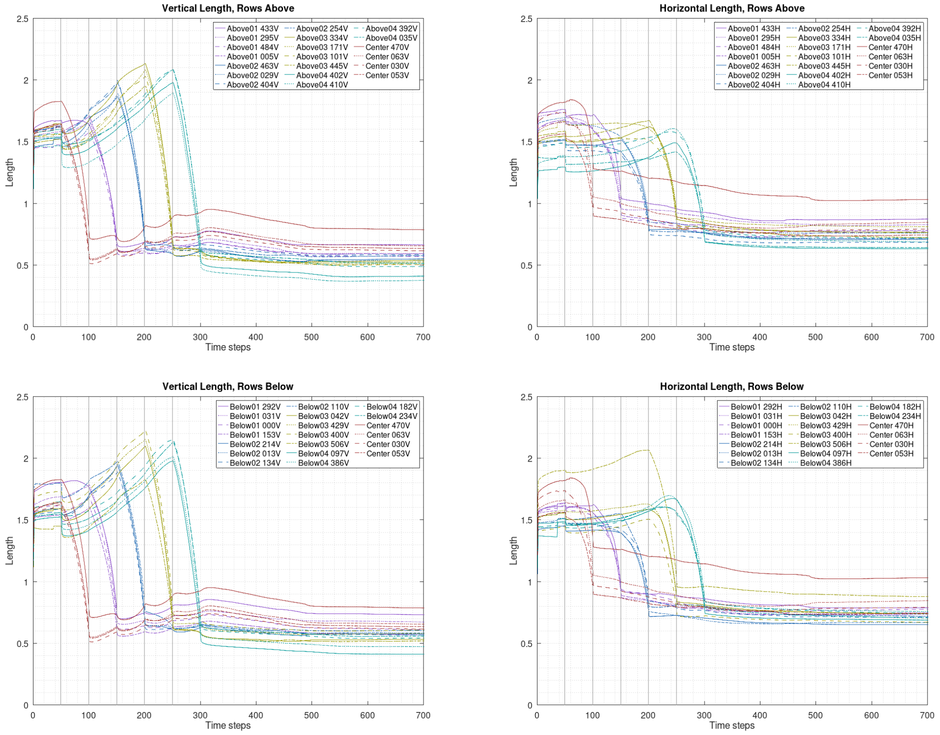

Fig 2. S1 Graphs
The left graphs give the area length vertically measured between 2 vertices, in the top figure the rows above the center rows are visualized and in the bottom figure, the rows below the center row are visualized. The right graphs give the horizontal measurements between two vertices. Top graph: again rows above the center row, bottom graph: rows below the center row. The lines are colored according to the colors used in Fig1.
