# Supplementary material for: Studying gastrulation by invagination: The bending of a cell sheet by mechanical cell properties using 3D deformable cell based simulations
Source: PLoS Comput Biol. 2025 Jun 25;21(6):e1013151. doi: 10.1371/journal.pcbi.1013151 (PMC12194075; doi:10.1371/journal.pcbi.1013151)
Supplement: S1 Table — Parameters used for 3D simulations in Figs 4–5. (PDF) [file pcbi.1013151.s011.pdf]

S1 Table

S1 Table: Parameters used for 3D simulations in Figs 4-5 Main text

**Parameters**	Fig 4A-C	Fig 4D-G	Fig 5A	Fig 5B	Fig 5C	Fig 5D	Fig 5E
Total number of cells	83	256	32-1024	32-1024	32-1024	32-256	256
Number of endoderm cells		36,58					36
Apical region endoderm	0-30%	0-30%	0-30%	0-30%	0-30%	0-40%	0-30%
Lateral region endoderm	30-70%	30-70%	30-70%	30-70%	30-70%		30-70%
Basal region endoderm	70-100%	70-100%	70-100%	70-100%	70-100%	40-100%	70-100%
Cell Stiffness apical k	0.5	0.5	0.5	0.5	1	1.4, 1.5	1
Cell Stiffness lateral k	0.5	0.5	0.5	0.5	0.5		0.5
Cell Stiffness basal k	0.5	0.5	0.5	0.5	0.1	0.35-0.1	0.1
Adhesion region	20-65%	20-65%	20-65%	20-65%	20-65%	20-65%	20-65%
Adhesion strength k	1.2	1.2	0.8	0.8	0.8	0.8	0.8
Constriction region	0-50%	0-50%	0-50%	0-50%	0-50%	0-50%	0-50%
Constriction factor	0.1	0.1	0.1	0.05	0.05	0.1	0.1
Time interval constriction	100	500	500	500	500	500	500

Table notes:
Total number of cells: Total number of cells in blastula.
Number of endoderm cells: Number of endodermal cells in blastula.
Apical region endoderm: Region of spherical cell that is appointed as apical area.
Lateral region endoderm: Region of spherical cell that is appointed as lateral area.
Basal region endoderm: Region of spherical cell that is appointed as basal area.
Cell stiffness apical: Cell stiffness of the apical region.
Cell stiffness lateral: Cell stiffness of the lateral region.
Cell stiffness basal: Cell stiffness of the basal region.
Adhesion region: Region of the spherical cell that can adhere to another cell.
Adhesion strength k: The force (k) that is put on the adhesion spring to keep the adhered cells together.
Constriction region: Region of the spherical cell that can constrict.
Constriction factor: The new edge rest length that the appointed region tries to become.
Time interval constriction: The duration time that it takes for an edge to constrict to its new edge length.
