# Supplementary material for: Studying gastrulation by invagination: The bending of a cell sheet by mechanical cell properties using 3D deformable cell based simulations
Source: PLoS Comput Biol. 2025 Jun 25;21(6):e1013151. doi: 10.1371/journal.pcbi.1013151 (PMC12194075; doi:10.1371/journal.pcbi.1013151)
Supplement: S2 Table — Parameters used for 3D simulations in Figs 6–7. (PDF) [file pcbi.1013151.s012.pdf]

S2 Table

S2 Table: Parameters used for 3D simulations in Figs 6 and 7A-E Main text.

**Parameters**	Fig 6A	Fig 6B	Fig 7A	Fig 7B	Fig 7C	Fig 7D	Fig 7E
Total number of cells	512	512	256	256	256	256	256
Number of endoderm cells	144	87	64	67	69	71	73
Apical region endoderm	0-50%	0-20%	0-30%	0-30%	0-30%	0-30%	0-30%
Lateral region endoderm		20-60%, 20-80%	30-70%	30-70%	30-70%	30-70%	30-70%
Basal region endoderm	50-100%	80-100%	70-100%	70-100%	70-100%	70-100%	70-100%
Cell stiffness apical k	0.25-0.5	1.8	1	1	1	1	1
Cell stiffness lateral k		1.4, 0.5	0.5	0.5	0.5	0.5	0.5
Cell stiffness basal k	0.25-0.2	0.1	0.1	0.1	0.1	0.1	0.1
Adhesion region	20-60%	20-60%	20-65%	20-65%	20-65%	20-65%	20-65%
Adhesion strength k	0.75-1	0.8	0.8	0.8	0.8	0.8	0.8
Constriction region	0-50%	0-50%	0-50%	0-50%	0-50%	0-50%	0-50%
Constriction factor	0.1	0.07	0.1	0.1	0.1	0.1	0.1
Time interval constriction	500	500	500	500	500	500	500

Tale notes:
Total number of cells: Total number of cells in blastula.
Number of endoderm cells: Number of endodermal cells in blastula.
Apical region endoderm: Region of spherical cell that is appointed as apical area.
Lateral region endoderm: Region of spherical cell that is appointed as lateral area.
Basal region endoderm: Region of spherical cell that is appointed as basal area.
Cell stiffness apical: Cell stiffness of the apical region.
Cell stiffness lateral: Cell stiffness of the lateral region.
Cell stiffness basal: Cell stiffness of the basal region.
Adhesion region: Region of the spherical cell that can adhere to another cell.
Adhesion strength k: The force (k) that is put on the adhesion spring to keep the adhered cells together.
Constriction region: Region of the spherical cell that can constrict.
Constriction factor: The new edge rest length that the appointed region tries to become.
Time interval constriction: The duration time that it takes for an edge to constrict to its new edge length.
