# Supplementary material for: Studying gastrulation by invagination: The bending of a cell sheet by mechanical cell properties using 3D deformable cell based simulations
Source: PLoS Comput Biol. 2025 Jun 25;21(6):e1013151. doi: 10.1371/journal.pcbi.1013151 (PMC12194075; doi:10.1371/journal.pcbi.1013151)
Supplement: S4 Table — Parameters used for simulations in Fig 3, Figs A,B,D–G, S2 Appendix. (PDF) [file pcbi.1013151.s014.pdf]

S4 Table

S4 Table: Parameters used for Fig 3 (Main text), and Planar simulations Figs A-G S2 Appendix

**Parameters**	Fig 3 Main text	Fig A	Fig B	Fig D	Fig E	Fig F	Fig G
Total number of cells	1	64	64	64	64	64	64
Number of endoderm cells	0	18	26	18	18	18	8-26
Apical region endoderm	0-30%	0-40%	0-30%	0-40%	0-40%	0-40%	0-40%
Lateral region endoderm	30-70%		30-70%				
Basal region endoderm	70-100%	40-100%	70-100%	40-100%	40-100%	40-100%	40-100%
Cell stiffness apical k	0.5	1.8	1	1.8	1.8	0.4-1.8	1.8
Cell stiffness lateral k	0.5		0.5				
Cell stiffness basal k	0.5	0.1	0.1	0.1	0.1	0.1	0.1
Adhesion region		20-50%	20-65%	20-75%	20-60%, 20-90%	20-75%	20-75%
Adhesion strength k		1.5	0.7	1.5	1.5	1.5	1.5
Constriction region	0-50%	0-50%	0-50%	0-50%	0-50%	0-50%	0-50%
Constriction factor	0.05	0.05	0.05	0.05-0.3	0.05	0.05	0.05
Time interval constriction			500, 750, 1000	1000	1000	1000	1000

Table notes:
Total number of cells: Total number of cells in blastula.
Number of endoderm cells: Number of endodermal cells in blastula.
Apical region endoderm: Region of spherical cell that is appointed as apical area.
Lateral region endoderm: Region of spherical cell that is appointed as lateral area.
Basal region endoderm: Region of spherical cell that is appointed as basal area.
Cell stiffness apical: Cell stiffness of the apical region.
Cell stiffness lateral: Cell stiffness of the lateral region.
Cell stiffness basal: Cell stiffness of the basal region.
Adhesion region: Region of the spherical cell that can adhere to another cell.
Adhesion strength k: The force (k) that is put on the adhesion spring to keep the adhered cells together.
Constriction region: Region of the spherical cell that can constrict.
Constriction factor: The new edge rest length that the appointed region tries to become.
Time interval constriction: The duration time that it takes for an edge to constrict to its new edge length.
